# Genetic architecture of cherry leaf spot (*Blumeriella jaapii*) resistance in sour cherry (*Prunus cerasus* L.) uncovered by QTL analyses in a biparental population genotyped with the 6 + 9 K SNP array

**DOI:** 10.1093/hr/uhaf035

**Published:** 2025-02-03

**Authors:** Ofere Francis Emeriewen, Thomas Wolfgang Wöhner, Vincent Braun, Susan Schröpfer, Mirko Schuster, Andreas Peil, Henryk Flachowsky

**Affiliations:** Julius Kühn-Institut (JKI), Federal Research Centre for Cultivated Plants, Institute for Breeding Research on Fruit Crops, Pillnitzer Platz 3a, Dresden D-01326, Germany; Julius Kühn-Institut (JKI), Federal Research Centre for Cultivated Plants, Institute for Breeding Research on Fruit Crops, Pillnitzer Platz 3a, Dresden D-01326, Germany; Julius Kühn-Institut (JKI), Federal Research Centre for Cultivated Plants, Institute for Breeding Research on Fruit Crops, Pillnitzer Platz 3a, Dresden D-01326, Germany; University of Hohenheim, Landessaatzuchtanstalt (720), Fruwirthstr. 21, Stuttgart 70599, Germany; Julius Kühn-Institut (JKI), Federal Research Centre for Cultivated Plants, Institute for Breeding Research on Fruit Crops, Pillnitzer Platz 3a, Dresden D-01326, Germany; Julius Kühn-Institut (JKI), Federal Research Centre for Cultivated Plants, Institute for Breeding Research on Fruit Crops, Pillnitzer Platz 3a, Dresden D-01326, Germany; Julius Kühn-Institut (JKI), Federal Research Centre for Cultivated Plants, Institute for Breeding Research on Fruit Crops, Pillnitzer Platz 3a, Dresden D-01326, Germany; Julius Kühn-Institut (JKI), Federal Research Centre for Cultivated Plants, Institute for Breeding Research on Fruit Crops, Pillnitzer Platz 3a, Dresden D-01326, Germany

## Abstract

Sour cherry (*Prunus cerasus* L.) is an economically significant species in the Rosaceae family. Hitherto, there had been limited genetic and genomic resources to elucidate important horticultural traits in this species mainly because of the complex polyploid nature of its genome, a hybrid between *Prunus avium* and *Prunus fruticosa*. An important trait that has not been well studied in sour cherry is resistance to cherry leaf spot (CLS), caused by the fungus *Blumeriella jaapii*. This work took advantage of the RosBREED 6 + 9 K SNP array to study the genetic basis of CLS resistance and inheritance in sour cherry. We established an F_**1**_ segregating population by crossing two cultivars, ‘Schattenmorelle’ and ‘Pc 2’ and genotyped both parents and the progeny with the cherry 6 + 9 K SNP array and SSR markers. We evaluated both parents and progeny for resistance and susceptibility to CLS under field conditions. The applied marker systems facilitated the development of parental genetic maps, and the identification of two stable QTLs associated with CLS resistance, *CLSR_1f* in ‘Pc 2’ and susceptibility, *CLSS_1f*, in ‘Schattenmorelle’ explaining 40.9% and 21.5%, respectively, of the phenotypic variation within the population. The mechanism of resistance in sour cherry appears to be independent of the CLS resistance QTL, *CLSR_G4*, previously identified in *P. canescens,* as the *CLSR_G4*-QTL and associated allele were not identified. Based on our findings, we propose a two-gene model for CLS resistance in sour cherry involving a susceptibility QTL, which might explain why some *CLSR_G4*-resistant plants in previous studies were susceptible.

## Introduction

The sour cherry (*Prunus cerasus* L.) is an economically important species in the genus *Prunus*, grown mainly in North America, Eastern and Central Europe, and Asia [1]. It belongs to the Rosaceae family of plants, which includes other economically important tree fruit crops like sweet cherry (*Prunus avium* L.), peach (*Prunus persica* L. Batsch), pears (*Pyrus* spp.), and apples (*Malus* × *domestica* Borkh.). In 2020, the production value of sour cherry was $US 1.2 billion. In addition, 1.51 Million tons of sour cherry fruits were produced in the year 2021 globally (https://www.fao.org/faostat/en/#data). The German cherry production, i.e. sweet and sour cherry, for 2022–2023 marketing year was estimated at 54 700 metric ton (MT), which is 19% above the preceding 10-year period from 2012–2021 (USDA report available at https://gain.fas.usda.gov/#/search).

*P. cerasus* (2n = 4x = 32) is a segmental allotetraploid [2], which originated from hybridization between the diploid *P*. *avium* (2n = 2x = 16) and the tetraploid ground cherry *P*. *fruticosa* Pall. (2n = 4x = 32) [3–5]. Therefore, the genome of *P*. *cerasus* is characterized by the presence of *P*. *avium* and *P*. *fruticosa* subgenomes [2]. This complex polyploidy and the hitherto lack of genomic resources have affected studies aimed at the genetic basis of several important horticultural traits in sour cherry. Hence, prior to the development of genomic resources specific for cherry [2, 3, 6, 7], genetic studies relied on the synteny between cherry and peach sequences and other *Prunus* species [8–12]. Although genetic maps have been developed for sweet and sour cherry using different marker systems [13–16], the recently developed 6 + 9 K single nucleotide polymorphisms (SNP) array [12], offers the opportunity for creation of high-density genetic maps. In the first instance, only a third of SNP markers from the first developed 6 K SNP array were informative with many genomic regions not covered [10]. Vanderzande *et al.* [12] then added the 9 K SNP array to provide for an increased genomic coverage and genetic resolution using reliable and polymorphic SNPs from sweet cherry, the two subgenomes of sour cherry as well as cherry organelle genome.

High-density genetic maps are important tools in genetic studies and to date, only the study of Stegmeir *et al.* [17] reports a dense genetic map in sour cherry to study the genetic basis of cherry leaf spot (CLS) resistance in sour cherry by means of five F_1_ sour cherry populations. CLS, caused by the fungal pathogen *Blumeriella jaapii* (Rehm), is increasingly becoming a major disease in cherries causing chlorotic and necrotic leaf spots and ultimately defoliation. Infection typically starts in middle of spring following deposition of spores by the fungus, which then leads to the production of conidia in the summer with favourable conditions [17]. Cherries, sweet and sour, are typically susceptible to the disease with variable degrees of susceptibility and cultivar-dependent resistance/tolerance, although *P*. *canescens* (Bois) S. Ya. Sokolo, the gray-leaf cherry, is known to be resistant [17]. Therefore, finding resistant or highly tolerant cultivars and introgressing resistance from such sources is increasingly important in breeding programmes especially due to the negative influence of climate change and global warming, which appears to favour the pathogen, as well as a decreasing availability of fungicides application by legal authorities [18]. A major CLS-resistance QTL (*CLSR_G4*) was previously identified on Chromosome 4 in *P. canescens* and validated in sour cherry introgression material [17]. Interestingly, Stegmeir *et al.* [17] proposed a two-gene model for the genetic control of CLS resistance in sweet and sour cherry. However, only the QTL on G4 could be identified in their study with the second putative QTL remaining elusive, which was probably due to the limited population size [17]. Furthermore, it remains unclear whether the CLS reactions in sweet and sour cherry are resistance, partial resistance or tolerance because according to Sjulin *et al.* [19], high resistance was not found but varying severity of defoliation in the field and number of days for 50% of infection to occur in the greenhouse were observed in cherry.

The aim of this work was to study the genetic basis of CLS resistance in sour cherry using a genetic mapping approach in a biparental population derived by crossing two cultivars of sour cherry, ‘Schattenmorelle’ and ‘Pc 2.’

## Results

### SNP application and analyses

We analysed 13 559 SNPs in total on both parents. Data analyses with the autopolyploid module of GenomeStudio software (illumina) showed that 1310 SNPs representing 9.7% were informative and could be used for further analyses. The remaining 12 249 SNPs, which represent 90.3% were either homozygous or failed SNPs, and therefore non-informative. Further, 236 SNPs (lm × ll) were polymorphic in only ‘Schattenmorelle’ whereas 330 SNPs (nn × np) were polymorphic in only ‘Pc 2’, and 744 SNPs (hk × hk) were polymorphic in both parents.

**Table 1:** Characteristics of obtained linkage maps for `Schattenmorelle' and ‘Pc 2’ and number of markers representing the subgenomes *P. avium* (a) and *P. fruticosa* (f) or in the event only one linkage map was calculated (a&f)

	`Schattenmorelle'						`Pc 2'					
*P. cerasus*	a	cM	f	cM	a&f	cM	a	cM	f	cM	a&f	cM
LG1	104	114.6	60	64.3			91	209.121	148	112.2		
LG2	61	97.8	78	115.9			62	140.256	81	84.9		
LG3	51	95.8	57	41.5			58	107.12	72	62.7		
LG4	58	98.0	68	44.9			58	86.623	84	88.5		
LG5	54	87.3	74	55.3			63	106.579	87	82.5		
LG6	33	92.0	91	77.6							96	86.2
LG7	65	96.0	59	89.2			65	90.85	58	83.3		
LG8	47	94.2	33	76.8			50	97.195	34	98.8		

With the exception of digits under ‘cM’, other digits are number of markers in each LG. LG = linkage group, cM = CentiMorgan.

### S‌SR application

Forty-four *Prunus* SSR markers (Supplemental data 1) applied to the parents and progeny produced multiple alleles in ‘Schattenmorelle’ and ‘Pc 2’. In general, 41 markers were polymorphic in both or each parent. Of these 41 SSR markers, 30 produced alleles, which were uniquely polymorphic in each parent resulting in 49 alleles in ‘Schattenmorelle’ and 71 alleles in ‘Pc 2’. Twenty-six SSRs produced 38 alleles that were polymorphic in both parents. Four F_1_ individuals were removed from the dataset due to the expression of alleles of SSR markers, which were not present in either parent and as such, these individuals were adjudged to be out crossers. One progeny was removed from mapping analyses because of numerous missing marker data. Thus, the genetic maps were created using marker data of 202 F_1_ individuals.

### Genetic maps represented the subgenomes of *P. cerasus*

One thousand and sixty-six loci (1066) used to create the genetic map of ‘Schattenmorelle’, resulted in 16 linkage groups (LGs). Similarly, 1202 loci resulted in the assignment of 16 LGs for ‘Pc 2’. For both parental maps (Supplemental data 2), it was possible to assign two groups per LG that represent chromosomes of the *P. avium* and *P. fruticosa* subgenomes of *P. cerasus* (Table 1)*.* The exception was LG6 of ‘Pc 2’ where only one group was assigned (Table 1). The characteristics of the assigned LGs is shown in Table 1. Linkage groups belonging to the subgenome of *P. avium* were designated with LG_a, whereas LGs belonging to the subgenome of *P. fruticosa* were designated with LG_f. For the respective parental maps, it was possible to distinguish the *P. avium* subgenome from the *P. fruticosa* subgenome because markers originally developed from *P. avium* were the majority (Table S1). However, for LGs that represent the *P. fruticosa* subgenome, *P. avium*-derived markers competed in almost a balanced ratio with *P. fruticosa*-derived markers (Table S1). Nevertheless, the lack of competition of *P. fruticosa*-derived markers in the LGs assigned to *P. avium* was a strong indication that these groups indeed represented the *P. avium* subgenome. The collinearity between the genetic position and the physical position on the ‘Schattenmorelle’ genome sequence [2] was moderate to high (Supplemental data 3).

### Variation in resistance and susceptibility levels between ‘Pc 2’ and ‘Schattenmorelle’

**Figure 1:**
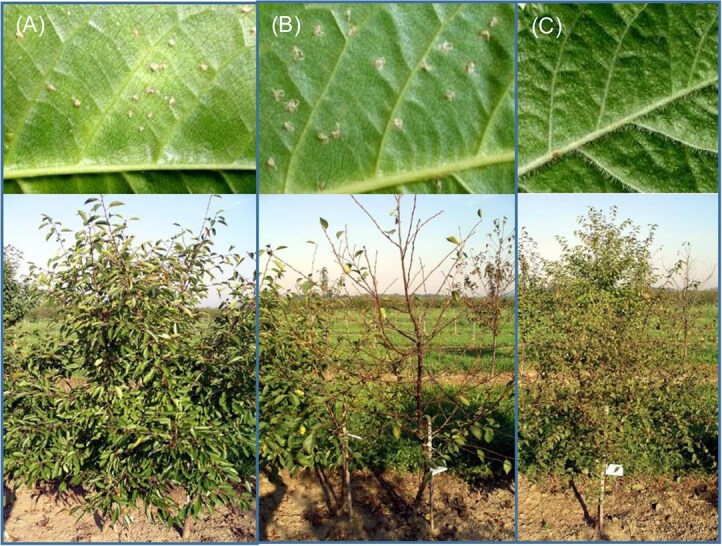
The reaction to CLS for the genotypes 'Pc 2′ (A), 'Schattenmorelle' (B), and *P. canescens* (C) after spray inoculation in the laboratory (upper pictures) in June, and in the field during natural infection at the end of August (lower pictures). The respective score type for laboratory reactions according to Wharton *et al.* [20] were as follows, 'Pc 2′: 4, 'Schattenmorelle': 4 and *P. canescens*: 1. Crown defoliation in the field evaluation were determined as follows (scoring value), 'Pc 2′: low to moderate (3), 'Schattenmorelle': very high (9) and, *P. canescens*: no (1).

A lab-performed detached leaf inoculation test of the both parents and *P*. *canescens* resulted in strong CLS symptom development on ‘Schattenmorelle’ and ‘Pc 2’ (>20 mature and sporulating acervuli). According to the scoring of Wharton *et al.* [20], the reaction type score determined for ‘Schattenmorelle‘ and ‘Pc 2’ was 4 whereas the score determined for *P*. *canescens* was 1 (Fig. 1).

**Figure 2:**
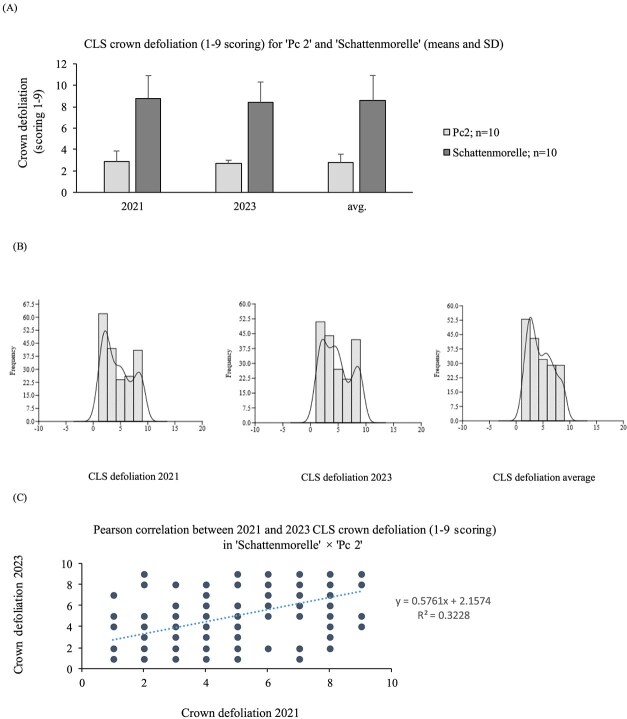
Statistics analysis for CLS defoliation scores in ‘Schattenmorelle’, ‘Pc 2’ and progenies. (**A**) means of scores for CLS defoliation calculated from 10 trees each per cultivar in 2021, 2023 and for both years with error bars (standard deviation). **(B)** Histogram plots indicating bimodal and left skewed distribution of CLS defoliation scores for the progenies in 2021, 2023 and the mean between both years, (**C**) Pearson correlation analysis between CLS defoliation scores between 2022 and 2023, r = coefficient of correlation.

A score of 1 was considered resistance whereas >3 was considered highly susceptible [20]. The CLS reactions of ‘Schattenmorelle’ and ‘Pc 2’ was further evaluated in the field on 10 trees each in 2021 and 2023. In general, ‘Pc 2’ developed significantly fewer symptoms of CLS in the field in both years of evaluation (Fig. 2A), in contrast to artificial inoculation trials in the lab. We observed symptoms in the field first on ‘Schattenmorelle’, and symptom progression resulted in complete defoliation by September 1^st^ in both years of evaluation. Symptom development on ‘Pc 2’ started much later and its progress was significantly slower resulting in a significantly lower crown defoliation in comparison to ‘Schattenmorelle’ (Fig. 2A). The average mean value (2021 and 2023) of ‘Pc 2’ was 2.8 (SD ± 1.2) and for ‘Schattenmorelle’ 8.6 (SD ± 1.3). The difference between the mean values of 2021 and 2023 was 0.2 for ‘Pc 2’ and 0.4 for ‘Schattenmorelle’. Based on this investigation accession ‘Pc 2’ was determined as having a mechanism to withstand CLS and ‘Schattenmorelle’ as highly susceptible. No symptoms were observed on *P. canescens* in the field evaluations.

The progenies of ‘Pc 2’ and ‘Schattenmorelle’ showed a variation of the average mean value from 1 to 9 (Fig. 2B). The population mean was calculated as 4.5 (SD ± 2.6) in 2021 and 4.7 (SD ± 2.6) in 2023. Plotting the trait into a histogram resulted in a bimodal distribution for 2021 and 2023. A Pearson correlation analysis (r = 0.57) indicates a correlation between both years and datasets (Fig. 2C).

**Table 2:** QTL mapping results from the linkage group 1f of cultivar ‘Pc 2’ (markers with highest LOD are indicated in bold)

marker	position in cM	2021 (GW: 9.8)				2023 (GW: 6.6)				avg. (GW: 8.7)			
		LOD	%-PVE	KW-value	p-value	LOD	%-PVE	KW-value	p-value	LOD	%-PVE	KW-value	p-value
1A_9415438	17.761	3.94	9.1	17.043	******	4.27	10.2	19.264	*******	5.42	12.8	22.4	*******
1F_10061954	17.909	4.74	10.9	16.832	******	4.96	11.9	18.274	******	6.25	14.7	21.167	*******
1A_10585398	18.1	4.65	10.7	16.154	******	4.88	11.7	17.563	******	6.12	14.4	20.324	*******
1A_13676278	23.984	3.38	7.9	17.167	******	2.61	6.3	13.4	****	3.68	8.8	16.43	******
1F_14899327	30.586	9.69	21.7	21.612	*******	5.63	13.6	23.512	*******	9.06	21.1	25.645	*******
**CPSCT027**	32.051	7.8	17.3	27.363	*******	**6.6**	**15.5**	**21.257**	*******	8.78	19.9	29.132	*******
1A_22393410	32.267	7.7	17.1	26.006	*******	5.65	13.4	17.943	*******	8.03	18.4	25.917	*******
1F_21818756	32.299	7.72	17.1	26.712	*******	5.65	13.4	17.831	*******	8.04	18.4	26.137	*******
**1F_13920852**	32.644	9.02	19.7	24.351	*******	6.55	15.3	24.578	*******	**9.2**	**20.7**	**27.04**	*******
1F_25579028	35.759	7.76	17.5	25.362	*******	4.44	11.1	12.831	******	7.14	17	21.681	*******
1F_24965573	36.183	8.04	18.2	26.92	*******	4.62	11.8	12.73	******	7.37	17.7	22.322	*******
**1F_25143168**	**36.183**	**10.04**	**40.9**	**27.622**	*******	4.36	24.9	12.65	******	7.98	33.8	22.526	*******
pchgms3_L3	39.43	7.78	18.2	23.038	*******	3.55	8.7	14.312	******	6.94	16.8	21.011	*******
1F_26283430	40.404	8.31	19.1	24.555	*******	3.11	7.8	11.72	*****	6.68	16.1	19.821	*******
1A_35024409	65.064	4.52	10.4	18.915	*******	3.5	8.5	13.229	******	4.99	11.8	19.211	*******
1F_35583350	66.092	3.91	9.1	15.645	*******	2.95	7.2	10.445	****	4.15	10	15.388	*******
BPPCT016_L2	70.306	3.19	7.4	13.216	******	2.02	5	8.601	****	3.28	7.9	13.343	******
1A_37026940	72.099	2.87	6.7	11.996	*****	1.84	4.5	7.534	***	2.69	6.5	10.711	****

GW – genome wide LOD threshold; cM – centi-Morgan; LOD – logarithm of odd, PVE – phenotypic variation explanation, KW – Kruskal Wallis value; p-value – less than 0.05 = ^*^, less than 0.01 = ^**^, less than 0.001 = ^***^, n asterisks indicate higher significance levels; avg. – averaged QTL results obtained by the analysis of means from 2021 and 2023

Table contains only a selection of markers mapped to LG1f, which represent the progression of significance levels along the linkage group

### QTL analyses identifies putative resistance and susceptibility QTLs

**Figure 3:**
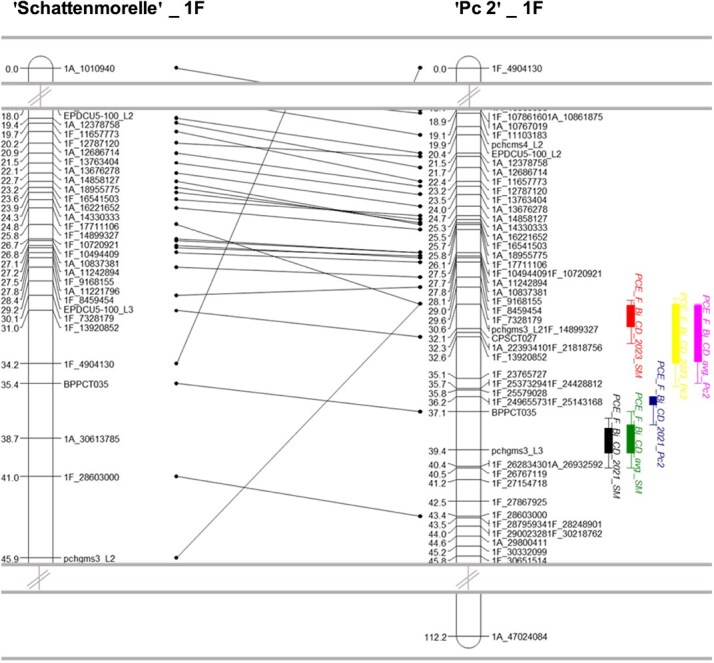
QTL region on linkage group 1F of 'Schattenmorelle' and 'Pc 2′. The colour bars indicate the QTL regions within the 1 and 2 LOD intervals for the averaged data and the years 2021 and 2023. Whisker bars with SM indicate the QTL interval in Schattenmorelle_1F group, whereas whisker bars with 'Pc 2′ indicate the QTL interval in Pc2_1F. Connection lines shows markers in common between both maps.

**Figure 4:**
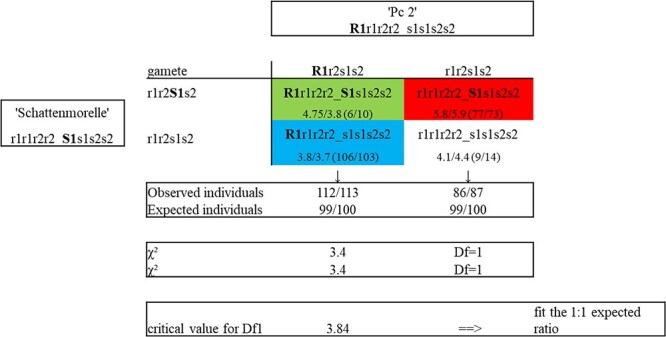
Gene model for CLS resistance and susceptibility in the sour cherry using a 'Schattenmorelle' × 'Pc 2′ biparental population. One half of the progeny have the dominant allele for CLS-resistance R1 for the QTL *CLSR*_1f. The other half have the dominant allele for CLS-high susceptibility S1 for the QTL *CLSS*_1f. The Punnett square shows the obtained allele combination for the progeny. Under each allele combination, the means of crown defoliation scorings are depicted and the number of individuals is shown in brackets. The first value represents the marker combination 1F_25143168 (R) with 1A_30613785 (S) and the second value the combination CPSCT027 (R) with 1A_30613785 (S). Resistant individuals carry the R1 allele and showed a mean defoliation score of 3.8/3.7 (blue). Highly susceptible individuals carry the S1 allele and showed a mean defoliation of 5.8/5.9 (red). Moderate individuals carrying both dominant alleles R1 and S1 are depicted in green. The recessive alleles are depicted in white. Chi-square test revealed that the observed segregation for R1 fit a the expected 1:1 ratio.

We identified and mapped two QTLs to LG1f in ‘Pc 2’ and in ‘Schattenmorelle’ using the map data and field disease evaluation data of the F_1_ progeny. The markers within the QTL *CLSR_1f* region in ‘Pc 2’ with the highest LOD score were IF_25143168 at 36.2 cM in 2021, CPSCT027 at 32.1 cM in 2023, and IF_13920852 at 32.6 cM for the average of both years, with IF_25143168 explaining up to 40.0% of the phenotypic variance (Table 2). The 1- and 2-LOD intervals are given in Fig. 3. The associated SNP marker 1F_25143168 (RosBREED_snp_tart_1_25143168, A/G) and the microsatellite CPSCT027 (presence of 181 bp) were found to be dominant for tolerance to CLS (R_1_r_1_r_2_r_2_). Individuals carrying R_1_ obtained a mean value of 3.8 or 3.7, respectively (indicated with blue in Fig. 4). The markers within the QTL in ‘Schattenmorelle’, named *CLSS_1f* with the highest LOD score were 1A_30613785 (scaffold_1:29639838, A/G) at 38.7 cM in 2021, 1F_13920852 at 31 cM in 2023, with 1A_30613785 retaining the highest LOD score for the average of both years. The QTL explained up to 28% of the phenotypic variance (Table 3). The SNP marker 1A_30613785 (scaffold_1:29639838) was found to be dominant for high susceptibility to CLS (S_1_s_1_s_2_s_2_). Individuals carrying S_1_ obtained a mean value of 5.8 or 5.9 respectively (indicated with red in Fig. 4). The QTLs were consistently detected using the data obtained in 2021 and 2023 as well as the average of both years (Table S2) with LOD values significantly higher than the calculated genome wide LOD threshold although the markers with the highest LOD changed between the observed years (Table 3). The QTL regions for resistance and susceptibility on ‘Pc 2′ and ‘Schattenmorelle’, respectively, overlap on Chromosome 1 as determined by the shared markers on their respective maps (Fig. 3). We identified two other inconsistent QTLs on LGs 2f and 3f in both parental genotypes. Whereas the QTL on LG2f was identified only with the 2021 data, the QTL on LG3f was identified only with the 2023 data (Table S2). Furthermore, we identified two unique QTLs with the 2021 data on LGs 1a and 7f only in the.‘Schattenmorelle’ map (Table S2). Based on these results, we propose a new gene model for CLS resistance susceptibility in sour cherry (Fig. 4). 

### CLS resistance in ‘Schattenmorelle’ × ‘Pc 2’ population is independent of *CLSR_G4* derived from *P. canescens*

*P. canescens* accession PRU0129 amplified a single 155 bp fragment for *CLSR_G4* allele-specific marker CLS028, which was found to be linked to the resistance haplotype of *P. canescens* [17]. Neither *P. maackii*, ‘Pc 2’, ‘Schattenmorelle’, nor any F_1_ offspring amplified the allele obtained from *P. canescens* (Table 4).

### Candidate gene identification

The BLAST of the markers within the interval (Table 2) resulted in the identification of their physical position between 13 177 509 bp and 31 939 109 bp. We found 41 resistance gene transcripts (types CC-NB-LRR, RLK, SPTMK, TIR-NB-LRR) located on Chromosome 1 in the subgenome of *P. fruticosa* (Supplemental data 4). A comparison of the amino acid identity results showed that 21 of the resistance gene transcripts are *P. fruticosa-*like, whereas six are *P. avium-*like. The remaining 14 transcripts could not be assigned to any of the subspecies of *P. cerasus.*

**Table 3:** QTL mapping results from the linkage group 1f of cultivar Schattenmorelle (marker with highest LOD are indicated in bold)

marker	position in cM	2021 (GW: 4.9)				2023 (GW: 4.5)				avg. (GW: 4.8)			
		LOD	%-PVE	KW	p-value	LOD	%-PVE	KW	p-value .	LOD	%-PVE	KW	p-value
1A_1010940	0	0.17	0.4	1.304	-	0.65	1.6	3.261	-	0.41	1	1.566	-
1A_2370183	1.776	0.22	0.5	1.249	-	0.74	1.8	3.104	-	0.57	1.4	2.162	-
1F_6110448	9.453	1.62	3.9	6.596	**	2.25	5.5	9.682	**	2.44	6	9.246	***
1F_5993141	9.483	1.62	3.9	6.583	**	2.25	5.5	9.708	**	2.44	6	9.27	***
1A_8440708	11.213	2.21	5.2	9.425	***	3.11	7.5	13.343	***	3.38	8.2	13.328	****
1F_8290152	12.809	2.46	5.8	10.876	****	3.05	7.4	13.597	****	3.46	8.4	14.091	*****
1A_9623424	15.452	3.38	7.9	15.234	******	4.08	9.8	18.17	******	4.86	11.6	20.131	*******
1A_10837381	27.069	5.22	12	20.681	*******	4.49	10.8	17.716	*******	5.58	13.3	20.564	*******
1A_11242894	27.193	5.21	12	20.722	*******	4.46	10.7	17.744	*******	5.56	13.2	20.522	*******
1F_9168155	27.513	5.53	12.6	22.022	*******	5.15	12.2	20.329	*******	6.24	14.6	23.359	*******
1A_11221796	27.819	5.04	12.1	4.231	**	4.58	11.6	3.571	**	5.6	14.1	4.452	**
1F_8459454	28.356	4.43	10.2	17.36	******	3.72	9	14.648	******	4.61	11	16.805	******
1F_7328179	30.064	3.11	7.4	11.359	****	2.86	7	11.268	****	3.43	8.4	11.953	****
**1F_13920852**	**30.959**	7.42	17.8	24.351	*******	**5.84**	**14.1**	**24.578**	*******	7.5	17.9	27.04	*******
1F_4904130	34.197	1.73	4.6	6.003	**	2.63	6.6	10.724	**	2.12	5.3	7.742	**
**1A_30613785**	**38.732**	**8.61**	**21.4**	**28.126**	*******	4.86	12.8	17.71	*******	**8.46**	**21.5**	**26.208**	*******
1A_32251525	53.021	6.81	16.6	15.58	******	3.49	9.3	6.504	******	6.68	17.3	13.141	****
1F_34299562	64.341	5.96	17.4	5.215	*	3.87	10.5	8.108	*	6.19	17.4	8.306	**

Table contains only a selection of markers mapped to LG1f, which represent the progression of significance levels along the linkage group

GW – genome wide LOD threshold; cM – centi-Morgan; LOD – logarithm of odd, PVE – phenotypic variation explanation, KW – Kruskal Wallis value; p-value – less than 0.05 = ^*^, less than 0.01 = ^**^, less than 0.001 = ^***^, n asterisks indicate higher significance levels; avg. – averaged QTL results obtained by the analysis of means from 2021 and 2023

**Table 4:** Allele sizes obtained for SSR marker CLS028 linked to *P. canescens* CLS resistance

**Genotypes**	**Allele sizes in base pair (bp)**				
*P. canescens* PRU029	155^*^				
					
`Schattenmorelle'	163	174	175	210	
					
`Pc 2'	162	175	179	196	206
					
^a^PiSa16-80.S71 _R	162	163	174	196	
					
^a^PiSa16-80.S16 _R	162	175	206	210	
					
^a^CK01 _02_144 _R	162	163	174	206	
					
^a^CK01_02_122_S	163	174	179	206	
					
^a^CK01_02_075_S	162	175	206	210	
					
^a^CK01_02_115 _S	174	175	179	206	
					
^a^CK01_02_120_S	175	206	210		
					
*P. maakii* PRU092	182	184	192		

a Progeny of `Schattenmorelle' × `Pc 2'; 155^*^bp allele linked to *P. canescens* resistance [17] R = resistant phenotype; S = susceptible phenotype

## Discussion

The application of the cherry 6 + 9 K array in this study facilitated the establishment of dense genetic parental maps for ‘Schattenmorelle’ and ‘Pc 2’, although the vast majority of the SNPs were not informative, which is consistent with previous studies [10, 12, 17, 22]. To our knowledge, the current study is the first involving the application of the RosBREED 6 + 9 K array in a sour cherry biparental population. We identified important SNPs and SSR markers significantly associated with resistance to *Blumeriella jaapii* in sour cherry using this population. Our study identified and mapped two major QTLs, *CLSR_1f* in ‘Pc 2’ and *CLSS_1f* in ‘Schattenmorelle’, linked to CLS resistance and susceptibility respectively. Our results also revealed that CLS resistance in ‘Pc 2’ is independent of the *CLSR_G4* resistance derived from *P. canescens*. The donor of resistance to *B. jaapii*, ‘Pc 2’, is a local sour cherry variety in Germany, with unknown parentage. Additionally, we identified candidate resistance genes in the QTL region on Chromosome 1 from the subgenome *P. fruticosa*. Our findings are consistent with previous research that utilized SNP arrays and SSR markers for genetic mapping in *Prunus* species. For instance, Peace *et al.* [10] developed a genome-wide 6 K SNP array for sweet and sour cherry, demonstrating the utility of SNP arrays in genetic studies. Similarly, Vanderzande *et al.* [12] improved the cherry SNP array, further highlighting its cost-effectiveness for genetic analysis in cherry breeding. Other studies, such as those by Calle *et al.* [22] and Guajardo *et al.* [14], have successfully used SNP arrays for QTL mapping in cherry species, reinforcing the relevance of our approach. The identification of *CLSR_1f* and *CLSS_1f* QTLs provides valuable markers for breeding programs aiming to enhance and introgress CLS resistance in sour cherries. The practical application of these findings could lead to the development of new cultivars with improved disease resistance, reducing the economic impact of CLS on cherry production. This aligns with previous studies that have identified QTLs associated with disease resistance in cherries [17]. The application of SNP and SSR markers in our study, although effective, encountered challenges such as a high number of monomorphic and low polymorphic markers. This issue is common in genetic studies using arrays, as noted by Peace *et al.* [10] and Vanderzande *et al.* [12], and reflects the need for high quality, polymorphic markers for effective mapping. Although we assigned subgenomes based on linkage mapping with the SNPs positions generated by Vanderzande *et al.* [12], the actual subgenomes / chromosomes where the SNPs map is speculative and debatable. The 6 + 9 k SNP array developed by Vanderzande *et al.* [12] contains SNPs originally obtained from *P. avium, P. cerasus* and *P. fruticosa* with a physical position according to *P. persica*. Additionally, SSR markers from other *Prunus* species, particularly *P. persica*, were successfully used in our study, demonstrating their transferability and utility in sour cherry genetic mapping [23]. Due to the synteny of these *Prunus* species, it is possible that SNPs developed from a donor applied in an allotetraploid species like *P. cerasus* genetically map in the corresponding homeolog subgenome. Using the allopolyploid module in GenomeStudio for genetic map construction yielded outcomes similar to those obtained through traditional methods, indicating its efficacy in tetraploid species like sour cherry. This genetic map for ‘Schattenmorelle’ and ‘Pc 2’ provides a valuable resource for future genetic studies and breeding programs. Previous studies, such as Wang *et al.* [16] and Canli [24], have shown the importance of genetic maps in *Prunus* species, and our findings contribute to this growing body of knowledge.

The phenotyping results for *Blumeriella jaapii* resistance revealed differences between lab and field observations. ‘Pc 2’ exhibited tolerance under both conditions, while ‘Schattenmorelle’ showed high susceptibility. These differences can be attributed to the varying conditions in lab and field environments and the different times of symptom occurrence. Similar phenomena have been described in other host-pathogen systems, where slower pathogen growth is associated with resistance. The species-specific resistance in *P. cerasus* offers a faster adaptability for breeding purposes compared to *P. canescens* [20, 25]. The association between markers and phenotypes was reinforced through QTL mapping, identifying *CLSR_1f and CLSS_1f* as major QTLs associated with CLS resistance and susceptibility. Although we identified putative resistance gene transcripts within this region on the ‘Schattenmorelle’ subgenome *P. fruticosa* that possess known resistance gene domains including those with TIR-NB-LRR, CC-NB-LRR, SPTMKs and RLKs, a fine-mapping approach of the regions of interest would be required to pinpoint the genes exactly controlling resistance/susceptibility to *B. jaapii*.

Furthermore, our study extends the gene model for CLS resistance in sour cherry beyond what was previously described by Stegmeir *et al.* [17] in *P. canescens*. Stegmeir *et al.* [17] identified a major QTL, *CLSR_G4*, on G4, requiring the *P. canescens*-derived R haplotype for resistance. Stegmeir *et al.* [17], proposed a two-gene model to explain CLS resistance, where individuals must possess both the *P. canescens*-derived R haplotype and a second locus to be resistant. Andersen *et al.* [21] reinforced these findings by proposing a dominant two-gene model for *P. canescens*-derived resistance and speculated about a recessive genes controlling CLS tolerance from sweet cherry. Furthermore, these authors speculated about the possibility of a dominant susceptibility alleles from ground cherry. Our findings support this two-gene model and suggest a susceptibility QTL, which may explain why some plants carrying the *CLSR_G4* resistance were still susceptible in Stegmeir *et al.* [17]. This extended gene model enables a hypothesis that incorporates both the identified resistance and susceptibility loci, providing a more comprehensive understanding of CLS resistance mechanisms.

## Conclusion and perspective

The identification of key QTLs and candidate genes paves the way for the development of resistant cultivars, potentially transforming cherry breeding programs. We hypothesize that resistance and susceptibility mechanisms involve specific genes identified in the QTL region, which require further functional characterization. Future research should focus on validating the identified QTLs in larger and diverse populations. Functional studies on the candidate resistance genes identified on Chromosome 1 will provide deeper insights into their roles in disease resistance. For this, sequencing of the actual donor, ‘Pc 2’, would be crucial. Collaborative efforts with ongoing breeding programs can accelerate the development of resistant cultivars and improve the overall sustainability of sour cherry production.

## Materials and methods

### Plant material and DNA extraction

We established a segregating F_1_ population from a ‘Schattenmorelle’ × ‘Pc 2’ cross, two sour cherry cultivars, which led to the generation of 207 progeny individuals. This progeny is being maintained in the experimental orchards of the JKI in Dresden-Pillnitz (Germany) on their own root. The parental genotypes ‘Schattenmorelle‘, ‘Pc 2′ as well as the CLS resistant diploid *P. canescens* accession PRU0129 and another tetraploid species, *P. maackii* Rupr. accession PRU092 were cultivated in the greenhouse for initial CLS inoculation trials as grafted trees on 'Piku 1′ or 'Piku 3′ rootstocks and were later maintained in the orchard.

DNA was extracted from the parents and progeny using Qiagen DNeasy Plant Mini Kit (Qiagen, Hilden, Germany) according to the manufacturer’s instruction. Leaf or buds were collected, freeze-dried and 20 mg subsequently homogenized (20 Hz, 1.5 min, mixer mill MM300, Retsch®, Haan) with two metal beads in a 2 ml save-lock-tube. DNA was thereafter quantified with agarose gel electrophoresis (2 μl DNA, 2 μ 6x loading buffer, 6 μl ddH_2_O). Ten μl per sample were loaded and electrophoresis was performed with 140 Volt for 60 minutes with a standard λ-DNA row (5, 10, 20, 40, 50, and 100 ng DNA μl^−1^). The final DNA was diluted to 10 ng/μl and stored in -20°C until required PCR analyses. For SNP genotyping, nucleic acid was quantified on a Qubit 2.0 fluorometer using the Qubit™ dsDNA BR Assay kit (Invitrogen™). A final DNA concentration of 20 ng/μl was used for analysis.

**Table 5:** Marker segregation types corresponding to ‘lm × ll’, ‘nn × np’ (simplex and triplex) or ‘hk × hk’ (double simplex) and expected type in case of double reduction. Bold and underlined letters indicate the informative SNPs

segregation types	parent 1	parent 2	progeny	progeny (double reduction)
lm × ll	**A** BBB	BBBB	**A** BBB / BBBB	**AA** BB
	AAA**B**	BBBB	A**B**BB / AABB	**BB** BB
	**A** BBB	**A** AAA	AAAB / AABB	**AA** AA
	AAA**B**	AAAA	AAA**B** / AAAA	**BB** AA
nn × np	BBBB	**A** BBB	**A** BBB / BBBB	**AA** BB
	BBBB	AAA**B**	A**B**BB / AABB	BB**BB**
	AAAA	**A** BBB	AA**A**B / AABB	AA**AA**
	AAAA	AAA**B**	AAA**B** / AAAA	AA**BB**
hk × hk	**A** BBB	**A** BBB	**AA** BB / **A**BBB + **A**BBB / BBBB	**AAAA**
	AAA**B**	AAA**B**	AA**BB** /AAA**B** + AAA**B** / AAAA	**BBBB**

### CLS field phenotyping and *B*. *jaapii* inoculation

We scored disease symptom development of parental genotypes and the F_1_ progeny in the field in 2021 and 2023 (around the 1st September) with a scoring range of 1–9 for crown defoliation symptom, where 1 is highly resistant (all leaves without symptoms and on the tree) and 9 is highly susceptible (heavy infections, all leaves drop down). The obtained data were plotted as histogram using Past 4.10 [26]. Means and standard deviation were calculated with MS Excel. This data was subsequently used for QTL mapping analysis. Furthermore, both parents and *P. canescens* (PRU0129) were tested at two different time points (May and June) in the laboratory via detached leaf inoculation as described elsewhere [25]. CLS reaction types were determined as described by Wharton *et al.* [20]. Briefly, 0: green leaf without symptoms, 1: small lesions, chlorotic and/or necrotic points, 2: larger lesions with developing aerial mycelium and stunted sporulating lesions, 3: 2 to 10 sporulating lesions and 4: >11 sporulating lesions. Phenotyping was performed between 10 and 24 days post inoculation (dpi).

### RosBREED 6 + 9 K cherry SNP application and data analyses

Genotyping of both parents and the 207 progeny individuals was done with the 6 + 9 K SNP array previously developed for sweet and sour cherry [12]. For the parental genotypes, four replicates were genotyped.

The filtering parameters for genotyping with these SNPs were the same as previously reported [10, 12]. The SNP genotypes were analysed on GenomeStudio Data analyses software (Illumina Inc. 2010) using the autopolyploid module, considering that sour cherry is a segmental allopolyploid [27]. We used the recommended GenCall cut-off of 0.15, where samples with less than this value are not assigned genotypes. The autopolyploid module considers five possible genotypes for each SNP namely AAAA, AAAB, AABB, ABBB, and BBBB.

Parental replicates were checked for consistency of obtained dosages. Markers that lack consistency in the parents, with >10% missing data, with >3% false genotyped progeny and plastid markers were removed from the data set. The final marker set was sorted by segregation types which correspond to ‘lm × ll’, ‘nn × np’ (simplex and triplex) or ‘hk × hk’ (double simplex and double triplex) (Table 5). Based on the genotypic assignment of the parents, SNP markers were classified as polymorphic in each or both parents, monomorphic or failed markers. Polymorphic markers were used and tested for segregation distortion with chi-square test (χ^2^ test α = 0.05) and markers with distorted segregation were removed.

### Simple sequence repeats (SSR) marker application

Primers of microsatellite markers were sourced from literature [13, 23, 28–33]. SSR primers were applied to the population using the Type-It kit (Qiagen, Hilden, Germany) in a 10 μl volume, which included 2 μl of DNA (20 ng). PCR conditions were 95°C for 1 min 30 s, followed by 40 cycles of 95°C for 1 min, between 55 and 60°C for 1 min 30 s (depending on the primer annealing temperature) and 72°C for 30 s, and an extension at 60°C for 30 min. The PCR products were diluted 1:100 and 1 μl of the dilution was mixed with 8.95 μl of HiDi formamide (Applied Biosystems) and 0.05 μl of Liz 600 size standard (Applied Biosystems) in a total volume of 10 μl and denatured in a thermocycler at 94°C for 5 minutes. The products were thereafter genotyped on an ABI 3500xL Genetic Analyzer (Applied Biosystems, ThermoFisher Scientific, Darmstadt, Germany). Subsequently, the SSR fragments were visualised and annotated using GeneMapper™ software version 6 (ThermoFisher Scientific, Darmstadt, Germany). The SSRs were analysed as dominant presence/absence alleles. Polymorphic markers were also tested for segregation distortion as previously mentioned.

### Linkage map construction, QTL analyses, and resistance gene identification

Genetic maps were constructed with the aforementioned polymorphic SNP and SSR markers using JoinMap® Software version 5 [34]. A LOD up to 17 was used to calculate the linkage maps of the respective parents using the Kosambi mapping function. The parental maps obtained were compared using MapChart [35].

The genotypic data as well as phenotypic data of CLS field scoring for the F_1_ progeny were used to determine marker-phenotype association by Kruskal-Wallis analyses and for QTL analyses using MapQTL®5 [36]. LOD thresholds to determine significance of detected QTLs were calculated using permutation tests. Resistance gene models predicted and described by Wöhner and Emeriewen [37] for the genome sequence of 'Schattenmorelle' were explored for candidate genes within identified QTL regions.

### Candidate gene identification

Since there is no genome of ‘Pc 2’, we used marker data to search for putative candidate genes within the QTL region using the ‘Schattemorelle’ genome [2, 37]. Markers were blasted against the genome sequences to identify their physical positions. Subsequently, the region was used to extract the candidate transcripts which were already annotated and described in Wöhner and Emeriewen [37]. Amino acid identity (IAA) was obtained from Wöhner *et al.* [2] to determine the most likely origin of the resistance gene transcript as previously described.

## Supplementary Material

Web_Material_uhaf035

## Data Availability

The data underlying this article are available in the article and online supplementary material. The resistance donor, ‘Pc 2’, is available on request to the corresponding authors or on request at https://www.deutsche-genbank-obst.de/einfuehrung/zugangsbedingungen.
