# Supplementary material for: Genetic architecture of cherry leaf spot (Blumeriella jaapii) resistance in sour cherry (Prunus cerasus L.) uncovered by QTL analyses in a biparental population genotyped with the 6 + 9 K SNP array
Source: Hortic Res. 2025 Feb 3;12(5):uhaf035. doi: 10.1093/hr/uhaf035 (PMC11992334; doi:10.1093/hr/uhaf035)
Supplement: Web_Material_uhaf035 [file web_material_uhaf035.zip › Supplemental data 2.pptx]

## Slide 1
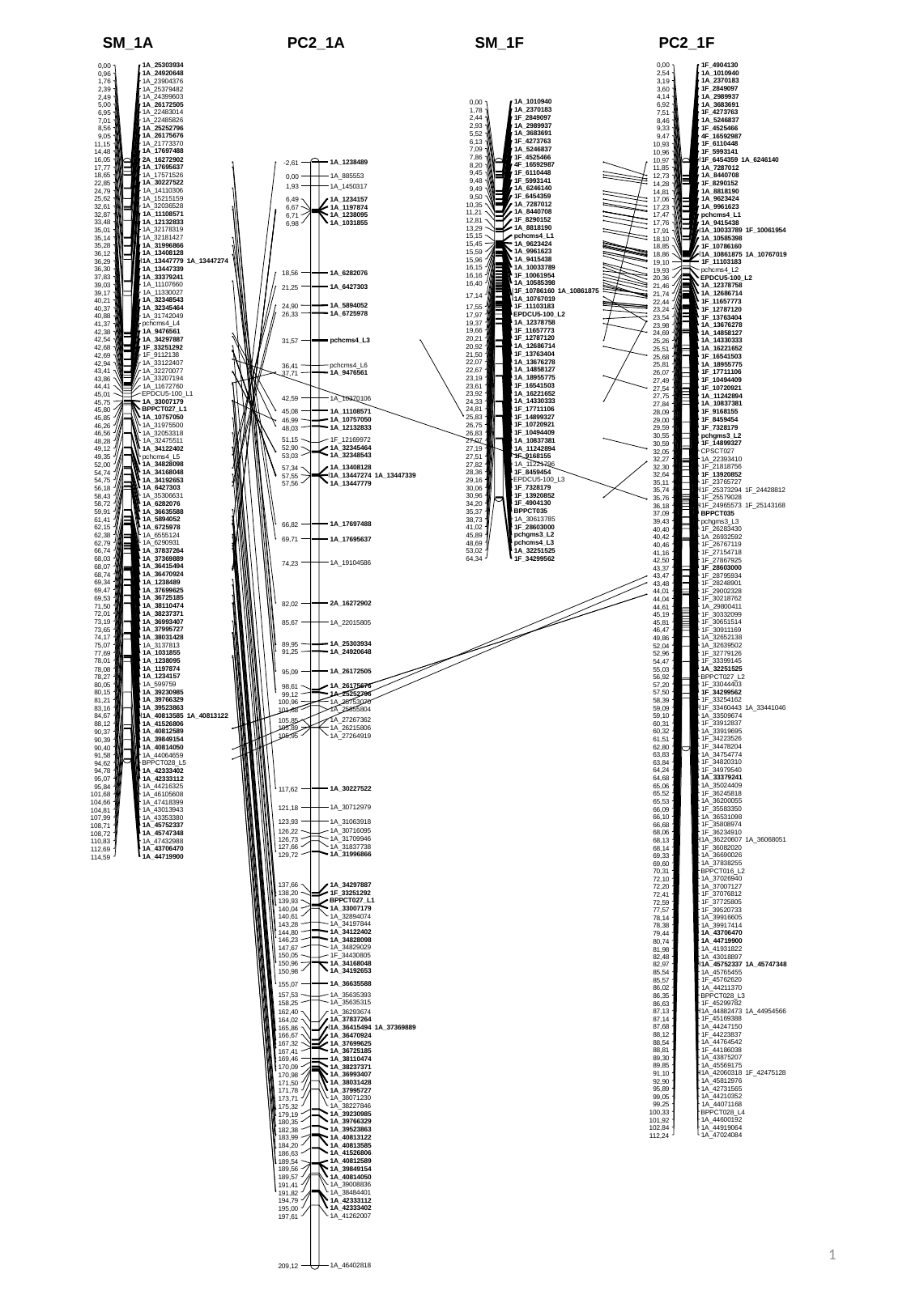

SM_1A
1A_25303934
0,00
1A_24920648
0,96
1A_23904376
1,76
1A_25379482
2,39
1A_24399603
2,49
1A_26172505
5,00
1A_22483014
6,95
1A_22485826
7,01
1A_25252796
8,56
1A_26175676
9,05
1A_21773370
11,15
1A_17697488
14,48
2A_16272902
16,05
1A_17695637
17,77
1A_17571526
18,65
1A_30227522
22,85
1A_14110306
24,79
1A_15215159
25,62
1A_32036528
32,61
1A_11108571
32,87
1A_12132833
33,48
1A_32178319
35,01
1A_32181427
35,14
1A_31996866
35,28
1A_13408128
36,12
1A_13447779
1A_13447274
36,29
1A_13447339
36,30
1A_33379241
37,83
1A_11107660
39,03
1A_11330027
39,17
1A_32348543
40,21
1A_32345464
40,37
1A_31742049
40,88
pchcms4_L4
41,37
1A_9476561
42,38
1A_34297887
42,54
1F_33251292
42,68
1F_9112138
42,69
1A_33122407
42,94
1A_32270077
43,41
1A_33207194
43,86
1A_11672760
44,41
EPDCU5-100_L1
45,01
1A_33007179
45,75
BPPCT027_L1
45,80
1A_10757050
45,85
1A_31975500
46,26
1A_32053318
46,56
1A_32475511
48,28
1A_34122402
49,12
pchcms4_L5
49,35
1A_34828098
52,00
1A_34168048
54,74
1A_34192653
54,75
1A_6427303
56,18
1A_35306631
58,43
1A_6282076
58,72
1A_36635588
59,91
1A_5894052
61,41
1A_6725978
62,15
1A_6555124
62,38
1A_6290931
62,79
1A_37837264
66,74
1A_37369889
68,03
1A_36415494
68,07
1A_36470924
68,74
1A_1238489
69,34
1A_37699625
69,47
1A_36725185
69,53
1A_38110474
71,50
1A_38237371
72,01
1A_36993407
73,19
1A_37995727
73,65
1A_38031428
74,17
1A_3137813
75,07
1A_1031855
77,69
1A_1238095
78,01
1A_1197874
78,08
1A_1234157
78,27
1A_599759
80,05
1A_39230985
80,15
1A_39766329
81,21
1A_39523863
83,16
1A_40813585
1A_40813122
84,67
1A_41526806
88,12
1A_40812589
90,37
1A_39849154
90,39
1A_40814050
90,40
1A_44064659
91,58
BPPCT028_L5
94,62
1A_42333402
94,78
1A_42333112
95,07
1A_44216325
95,84
1A_46105608
101,68
1A_47418399
104,66
1A_43013943
104,81
1A_43353380
107,99
1A_45752337
108,71
1A_45747348
108,72
1A_47432988
110,83
1A_43706470
112,69
1A_44719900
114,59
PC2_1A
1A_1238489
-2,61
1A_885553
0,00
1A_1450317
1,93
1A_1234157
6,49
1A_1197874
6,67
1A_1238095
6,71
1A_1031855
6,98
1A_6282076
18,56
1A_6427303
21,25
1A_5894052
24,90
1A_6725978
26,33
pchcms4_L3
31,57
pchcms4_L6
36,41
1A_9476561
37,71
1A_10370106
42,59
1A_11108571
45,08
1A_10757050
46,99
1A_12132833
48,03
1F_12169972
51,15
1A_32345464
52,90
1A_32348543
53,03
1A_13408128
57,34
1A_13447274
1A_13447339
57,55
1A_13447779
57,56
1A_17697488
66,82
1A_17695637
69,71
1A_19104586
74,23
2A_16272902
82,02
1A_22015805
85,67
1A_25303934
89,95
1A_24920648
91,25
1A_26172505
95,09
1A_26175676
98,61
1A_25252796
99,12
1A_25753070
100,96
1A_25855804
101,68
1A_27267362
105,85
1A_26215806
105,89
1A_27264919
105,95
1A_30227522
117,62
1A_30712979
121,18
1A_31063918
123,93
1A_30716095
126,22
1A_31709946
126,73
1A_31837738
127,66
1A_31996866
129,72
1A_34297887
137,66
1F_33251292
138,20
BPPCT027_L1
139,93
1A_33007179
140,04
1A_32894074
140,61
1A_34197844
143,28
1A_34122402
144,80
1A_34828098
146,23
1A_34829029
147,67
1F_34430805
150,05
1A_34168048
150,96
1A_34192653
150,98
1A_36635588
155,07
1A_35635393
157,53
1A_35635315
158,25
1A_36293674
162,40
1A_37837264
164,02
1A_36415494
1A_37369889
165,86
1A_36470924
166,67
1A_37699625
167,32
1A_36725185
167,41
1A_38110474
169,46
1A_38237371
170,09
1A_36993407
170,98
1A_38031428
171,50
1A_37995727
171,78
1A_38071230
173,71
1A_38227846
175,32
1A_39230985
179,19
1A_39766329
180,35
1A_39523863
182,38
1A_40813122
183,99
1A_40813585
184,20
1A_41526806
186,63
1A_40812589
189,54
1A_39849154
189,56
1A_40814050
189,57
1A_39008836
191,41
1A_38484401
191,82
1A_42333112
194,79
1A_42333402
195,00
1A_41262007
197,61
1A_46402818
209,12
SM_1F
1A_1010940
0,00
1A_2370183
1,78
1F_2849097
2,44
1A_2989937
2,93
1A_3683691
5,52
1F_4273763
6,13
1A_5246837
7,09
1F_4525466
7,86
4F_16592987
8,20
1F_6110448
9,45
1F_5993141
9,48
1A_6246140
9,49
1F_6454359
9,50
1A_7287012
10,35
1A_8440708
11,21
1F_8290152
12,81
1A_8818190
13,29
pchcms4_L1
15,15
1A_9623424
15,45
1A_9961623
15,59
1A_9415438
15,96
1A_10033789
16,15
1F_10061954
16,16
1A_10585398
16,40
1F_10786160
1A_10861875
17,14
1A_10767019
1F_11103183
17,55
EPDCU5-100_L2
17,97
1A_12378758
19,37
1F_11657773
19,66
1F_12787120
20,21
1A_12686714
20,92
1F_13763404
21,50
1A_13676278
22,07
1A_14858127
22,67
1A_18955775
23,19
1F_16541503
23,61
1A_16221652
23,92
1A_14330333
24,33
1F_17711106
24,81
1F_14899327
25,83
1F_10720921
26,75
1F_10494409
26,83
1A_10837381
27,07
1A_11242894
27,19
1F_9168155
27,51
1A_11221796
27,82
1F_8459454
28,36
EPDCU5-100_L3
29,16
1F_7328179
30,06
1F_13920852
30,96
1F_4904130
34,20
BPPCT035
35,37
1A_30613785
38,73
1F_28603000
41,02
pchgms3_L2
45,89
pchcms4_L3
48,69
1A_32251525
53,02
1F_34299562
64,34
PC2_1F
1F_4904130
0,00
1A_1010940
2,54
1A_2370183
3,19
1F_2849097
3,60
1A_2989937
4,14
1A_3683691
6,92
1F_4273763
7,51
1A_5246837
8,46
1F_4525466
9,33
4F_16592987
9,47
1F_6110448
10,93
1F_5993141
10,96
1F_6454359
1A_6246140
10,97
1A_7287012
11,85
1A_8440708
12,73
1F_8290152
14,28
1A_8818190
14,81
1A_9623424
17,06
1A_9961623
17,23
pchcms4_L1
17,47
1A_9415438
17,76
1A_10033789
1F_10061954
17,91
1A_10585398
18,10
1F_10786160
18,85
1A_10861875
1A_10767019
18,86
1F_11103183
19,10
pchcms4_L2
19,93
EPDCU5-100_L2
20,36
1A_12378758
21,46
1A_12686714
21,74
1F_11657773
22,44
1F_12787120
23,24
1F_13763404
23,54
1A_13676278
23,98
1A_14858127
24,69
1A_14330333
25,26
1A_16221652
25,51
1F_16541503
25,68
1A_18955775
25,81
1F_17711106
26,07
1F_10494409
27,49
1F_10720921
27,54
1A_11242894
27,75
1A_10837381
27,84
1F_9168155
28,09
1F_8459454
29,00
1F_7328179
29,59
pchgms3_L2
30,55
1F_14899327
30,59
CPSCT027
32,05
1A_22393410
32,27
1F_21818756
32,30
1F_13920852
32,64
1F_23765727
35,11
1F_25373294
1F_24428812
35,74
1F_25579028
35,76
1F_24965573
1F_25143168
36,18
BPPCT035
37,09
pchgms3_L3
39,43
1F_26283430
40,40
1A_26932592
40,42
1F_26767119
40,46
1F_27154718
41,16
1F_27867925
42,50
1F_28603000
43,37
1F_28795934
43,47
1F_28248901
43,48
1F_29002328
44,01
1F_30218762
44,04
1A_29800411
44,61
1F_30332099
45,19
1F_30651514
45,81
1F_30911169
46,47
1A_32652138
49,86
1A_32639502
52,04
1F_32779126
52,96
1F_33399145
54,47
1A_32251525
55,03
BPPCT027_L2
56,92
1F_33044403
57,20
1F_34299562
57,50
1F_33254162
58,39
1F_33460443
1A_33441046
59,09
1A_33509674
59,10
1F_33912837
60,31
1A_33919695
60,32
1F_34223526
61,51
1F_34478204
62,80
1A_34754774
63,83
1F_34820310
63,84
1F_34979540
64,24
1A_33379241
64,68
1A_35024409
65,06
1F_36245818
65,52
1A_36200055
65,53
1F_35583350
66,09
1A_36531098
66,10
1F_35808974
66,68
1F_36234910
68,06
1A_36220607
1A_36068051
68,13
1F_36082020
68,14
1A_36690026
69,33
1A_37838255
69,60
BPPCT016_L2
70,31
1A_37026940
72,10
1A_37007127
72,20
1F_37076812
72,41
1F_37725805
72,59
1F_39520733
77,57
1A_39916605
78,14
1A_39917414
78,38
1A_43706470
79,44
1A_44719900
80,74
1A_41931822
81,98
1A_43018897
82,48
1A_45752337
1A_45747348
82,97
1A_45765455
85,54
1F_45762620
85,57
1A_44211370
86,02
BPPCT028_L3
86,35
1F_45299782
86,63
1A_44882473
1A_44954566
87,13
1F_45169388
87,14
1A_44247150
87,68
1F_44223837
88,12
1A_44764542
88,54
1F_44186038
88,81
1A_43875207
89,30
1A_45569175
89,85
1A_42060318
1F_42475128
91,10
1A_45812976
92,90
1A_42731565
95,89
1A_44210352
99,05
1A_44071168
99,25
BPPCT028_L4
100,33
1A_44600192
101,92
1A_44919064
102,84
1A_47024084
112,24
1

## Slide 2
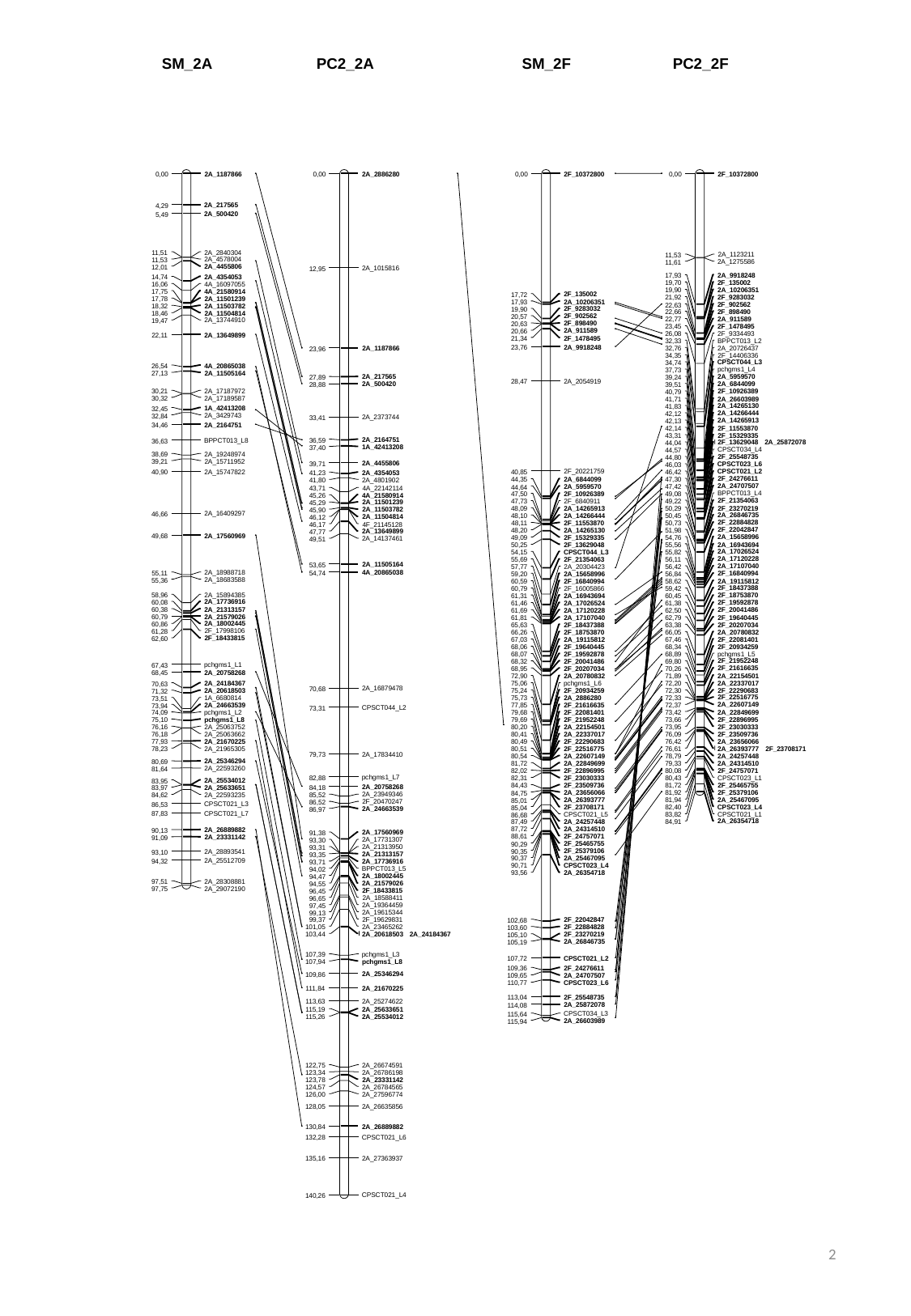

SM_2A
2A_1187866
0,00
2A_217565
4,29
2A_500420
5,49
2A_2840304
11,51
2A_4578004
11,53
2A_4455806
12,01
2A_4354053
14,74
4A_16097055
16,06
4A_21580914
17,75
2A_11501239
17,78
2A_11503782
18,32
2A_11504814
18,46
2A_13744910
19,47
2A_13649899
22,11
4A_20865038
26,54
2A_11505164
27,13
2A_17187972
30,21
2A_17189587
30,32
1A_42413208
32,45
2A_3429743
32,84
2A_2164751
34,46
BPPCT013_L8
36,63
2A_19248974
38,69
2A_15711952
39,21
2A_15747822
40,90
2A_16409297
46,66
2A_17560969
49,68
2A_18988718
55,11
2A_18683588
55,36
2A_15894385
58,96
2A_17736916
60,08
2A_21313157
60,38
2A_21579026
60,79
2A_18002445
60,86
2F_17998106
61,28
2F_18433815
62,60
pchgms1_L1
67,43
2A_20758268
68,45
2A_24184367
70,63
2A_20618503
71,32
1A_6680814
73,51
2A_24663539
73,94
pchgms1_L2
74,09
pchgms1_L8
75,10
2A_25063752
76,16
2A_25063662
76,18
2A_21670225
77,93
2A_21965305
78,23
2A_25346294
80,69
2A_22593260
81,64
2A_25534012
83,95
2A_25633651
83,97
2A_22593235
84,62
CPSCT021_L3
86,53
CPSCT021_L7
87,83
2A_26889882
90,13
2A_23331142
91,09
2A_28893541
93,10
2A_25512709
94,32
2A_28308881
97,51
2A_29072190
97,75
PC2_2A
2A_2886280
0,00
2A_1015816
12,95
2A_1187866
23,96
2A_217565
27,89
2A_500420
28,88
2A_2373744
33,41
2A_2164751
36,59
1A_42413208
37,40
2A_4455806
39,71
2A_4354053
41,23
2A_4801902
41,80
4A_22142114
43,71
4A_21580914
45,26
2A_11501239
45,29
2A_11503782
45,90
2A_11504814
46,12
4F_21145128
46,17
2A_13649899
47,77
2A_14137461
49,51
2A_11505164
53,65
4A_20865038
54,74
2A_16879478
70,68
CPSCT044_L2
73,31
2A_17834410
79,73
pchgms1_L7
82,88
2A_20758268
84,18
2A_23949346
85,52
2F_20470247
86,52
2A_24663539
86,97
2A_17560969
91,38
2A_17731307
93,30
2A_21313950
93,31
2A_21313157
93,35
2A_17736916
93,71
BPPCT013_L5
94,02
2A_18002445
94,47
2A_21579026
94,55
2F_18433815
96,45
2A_18588411
96,65
2A_19364459
97,45
2A_19615344
99,13
2F_19629831
99,37
2A_23465262
101,05
2A_20618503
2A_24184367
103,44
pchgms1_L3
107,39
pchgms1_L8
107,94
2A_25346294
109,86
2A_21670225
111,84
2A_25274622
113,63
2A_25633651
115,19
2A_25534012
115,26
2A_26674591
122,75
2A_26786198
123,34
2A_23331142
123,78
2A_26784565
124,57
2A_27596774
126,00
2A_26635856
128,05
2A_26889882
130,84
CPSCT021_L6
132,28
2A_27363937
135,16
CPSCT021_L4
140,26
SM_2F
2F_10372800
0,00
2F_135002
17,72
2A_10206351
17,93
2F_9283032
19,90
2F_902562
20,57
2F_898490
20,63
2A_911589
20,66
2F_1478495
21,34
2A_9918248
23,76
2A_2054919
28,47
2F_20221759
40,85
2A_6844099
44,35
2A_5959570
44,64
2F_10926389
47,50
2F_6840911
47,73
2A_14265913
48,09
2A_14266444
48,10
2F_11553870
48,11
2A_14265130
48,20
2F_15329335
49,09
2F_13629048
50,25
CPSCT044_L3
54,15
2F_21354063
55,69
2A_20304423
57,77
2A_15658996
59,20
2F_16840994
60,59
2F_16005866
60,79
2A_16943694
61,31
2A_17026524
61,46
2A_17120228
61,69
2A_17107040
61,81
2F_18437388
65,63
2F_18753870
66,26
2A_19115812
67,03
2F_19640445
68,06
2F_19592878
68,07
2F_20041486
68,32
2F_20207034
68,95
2A_20780832
72,90
pchgms1_L6
75,06
2F_20934259
75,24
2A_2886280
75,73
2F_21616635
77,85
2F_22081401
79,68
2F_21952248
79,69
2A_22154501
80,20
2A_22337017
80,41
2F_22290683
80,49
2F_22516775
80,51
2A_22607149
80,54
2A_22849699
81,72
2F_22896995
82,02
2F_23030333
82,31
2F_23509736
84,43
2A_23656066
84,75
2A_26393777
85,01
2F_23708171
85,04
CPSCT021_L5
86,68
2A_24257448
87,49
2A_24314510
87,72
2F_24757071
88,61
2F_25465755
90,29
2F_25379106
90,35
2A_25467095
90,37
CPSCT023_L4
90,71
2A_26354718
93,56
2F_22042847
102,68
2F_22884828
103,60
2F_23270219
105,10
2A_26846735
105,19
CPSCT021_L2
107,72
2F_24276611
109,36
2A_24707507
109,65
CPSCT023_L6
110,77
2F_25548735
113,04
2A_25872078
114,08
CPSCT034_L3
115,64
2A_26603989
115,94
PC2_2F
2F_10372800
0,00
2A_1123211
11,53
2A_1275586
11,61
2A_9918248
17,93
2F_135002
19,70
2A_10206351
19,90
2F_9283032
21,92
2F_902562
22,63
2F_898490
22,66
2A_911589
22,77
2F_1478495
23,45
2F_9334493
26,08
BPPCT013_L2
32,33
2A_20726437
32,76
2F_14406336
34,35
CPSCT044_L3
34,74
pchgms1_L4
37,73
2A_5959570
39,24
2A_6844099
39,51
2F_10926389
40,79
2A_26603989
41,71
2A_14265130
41,83
2A_14266444
42,12
2A_14265913
42,13
2F_11553870
42,14
2F_15329335
43,31
2F_13629048
2A_25872078
44,04
CPSCT034_L4
44,57
2F_25548735
44,80
CPSCT023_L6
46,03
CPSCT021_L2
46,42
2F_24276611
47,30
2A_24707507
47,42
BPPCT013_L4
49,08
2F_21354063
49,22
2F_23270219
50,29
2A_26846735
50,45
2F_22884828
50,73
2F_22042847
51,98
2A_15658996
54,76
2A_16943694
55,56
2A_17026524
55,82
2A_17120228
56,11
2A_17107040
56,42
2F_16840994
56,84
2A_19115812
58,62
2F_18437388
59,42
2F_18753870
60,45
2F_19592878
61,38
2F_20041486
62,50
2F_19640445
62,79
2F_20207034
63,38
2A_20780832
66,05
2F_22081401
67,46
2F_20934259
68,34
pchgms1_L5
68,89
2F_21952248
69,80
2F_21616635
70,26
2A_22154501
71,89
2A_22337017
72,20
2F_22290683
72,30
2F_22516775
72,33
2A_22607149
72,37
2A_22849699
73,42
2F_22896995
73,66
2F_23030333
73,95
2F_23509736
76,09
2A_23656066
76,42
2A_26393777
2F_23708171
76,61
2A_24257448
78,79
2A_24314510
79,33
2F_24757071
80,08
CPSCT023_L1
80,43
2F_25465755
81,72
2F_25379106
81,92
2A_25467095
81,94
CPSCT023_L4
82,40
CPSCT021_L1
83,82
2A_26354718
84,91
2

## Slide 3
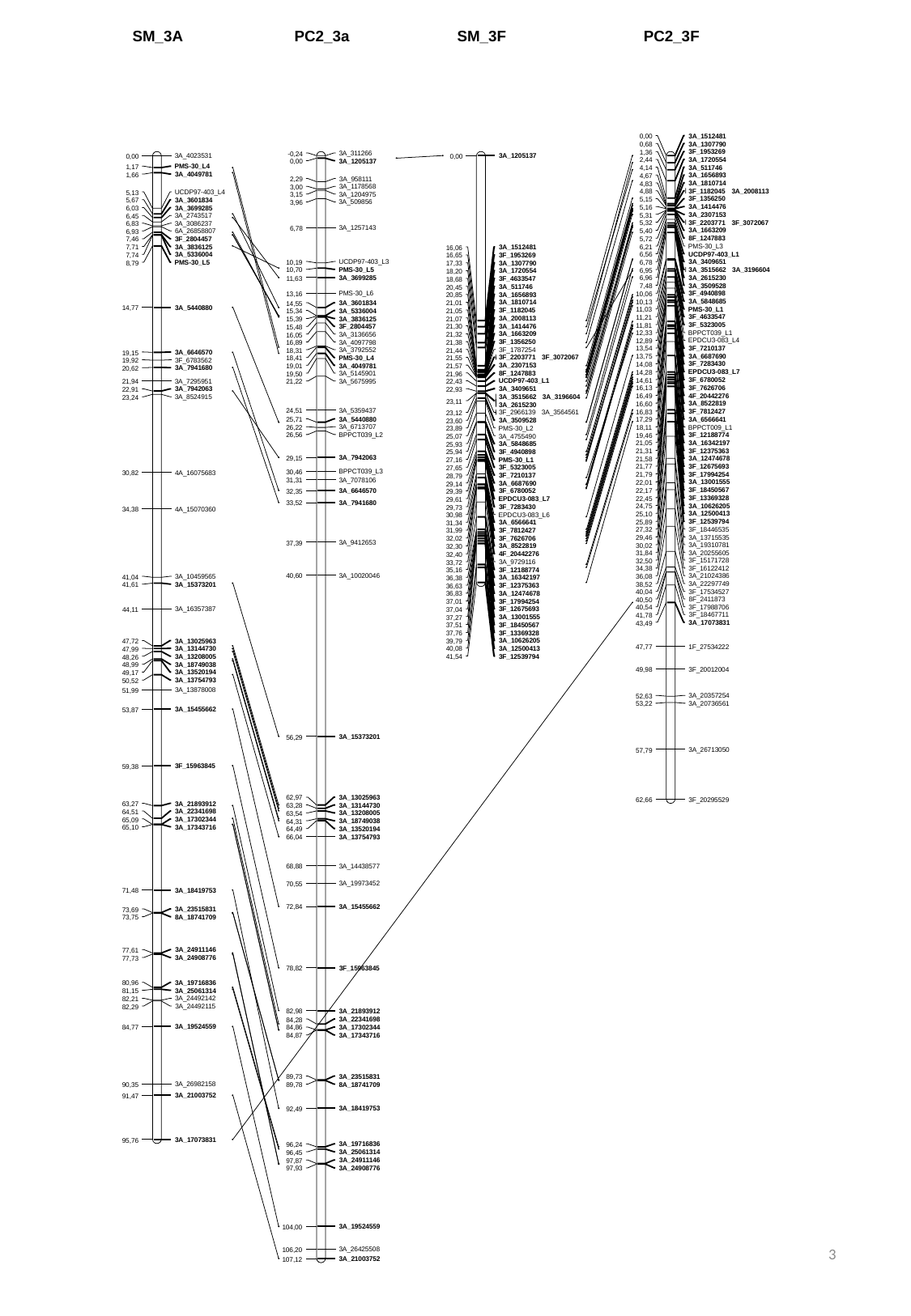

SM_3A
3A_4023531
0,00
PMS-30_L4
1,17
3A_4049781
1,66
UCDP97-403_L4
5,13
3A_3601834
5,67
3A_3699285
6,03
3A_2743517
6,45
3A_3086237
6,83
6A_26858807
6,93
3F_2804457
7,46
3A_3836125
7,71
3A_5336004
7,74
PMS-30_L5
8,79
3A_5440880
14,77
3A_6646570
19,15
3F_6783562
19,92
3A_7941680
20,62
3A_7295951
21,94
3A_7942063
22,91
3A_8524915
23,24
4A_16075683
30,82
4A_15070360
34,38
3A_10459565
41,04
3A_15373201
41,61
3A_16357387
44,11
3A_13025963
47,72
3A_13144730
47,99
3A_13208005
48,26
3A_18749038
48,99
3A_13520194
49,17
3A_13754793
50,52
3A_13878008
51,99
3A_15455662
53,87
3F_15963845
59,38
3A_21893912
63,27
3A_22341698
64,51
3A_17302344
65,09
3A_17343716
65,10
3A_18419753
71,48
3A_23515831
73,69
8A_18741709
73,75
3A_24911146
77,61
3A_24908776
77,73
3A_19716836
80,96
3A_25061314
81,15
3A_24492142
82,21
3A_24492115
82,29
3A_19524559
84,77
3A_26982158
90,35
3A_21003752
91,47
3A_17073831
95,76
PC2_3a
3A_311266
-0,24
3A_1205137
0,00
3A_958111
2,29
3A_1178568
3,00
3A_1204975
3,15
3A_509856
3,96
3A_1257143
6,78
UCDP97-403_L3
10,19
PMS-30_L5
10,70
3A_3699285
11,63
PMS-30_L6
13,16
3A_3601834
14,55
3A_5336004
15,34
3A_3836125
15,39
3F_2804457
15,48
3A_3136656
16,05
3A_4097798
16,89
3A_3792552
18,31
PMS-30_L4
18,41
3A_4049781
19,01
3A_5145901
19,50
3A_5675995
21,22
3A_5359437
24,51
3A_5440880
25,71
3A_6713707
26,22
BPPCT039_L2
26,56
3A_7942063
29,15
BPPCT039_L3
30,46
3A_7078106
31,31
3A_6646570
32,35
3A_7941680
33,52
3A_9412653
37,39
3A_10020046
40,60
3A_15373201
56,29
3A_13025963
62,97
3A_13144730
63,28
3A_13208005
63,54
3A_18749038
64,31
3A_13520194
64,49
3A_13754793
66,04
3A_14438577
68,88
3A_19973452
70,55
3A_15455662
72,84
3F_15963845
78,82
3A_21893912
82,98
3A_22341698
84,28
3A_17302344
84,86
3A_17343716
84,87
3A_23515831
89,73
8A_18741709
89,78
3A_18419753
92,49
3A_19716836
96,24
3A_25061314
96,45
3A_24911146
97,87
3A_24908776
97,93
3A_19524559
104,00
3A_26425508
106,20
3A_21003752
107,12
SM_3F
3A_1205137
0,00
3A_1512481
16,06
3F_1953269
16,65
3A_1307790
17,33
3A_1720554
18,20
3F_4633547
18,68
3A_511746
20,45
3A_1656893
20,85
3A_1810714
21,01
3F_1182045
21,05
3A_2008113
21,07
3A_1414476
21,30
3A_1663209
21,32
3F_1356250
21,38
3F_1787254
21,44
3F_2203771
3F_3072067
21,55
3A_2307153
21,57
8F_1247883
21,96
UCDP97-403_L1
22,43
3A_3409651
22,93
3A_3515662
3A_3196604
23,11
3A_2615230
3F_2966139
3A_3564561
23,12
3A_3509528
23,60
PMS-30_L2
23,89
3A_4755490
25,07
3A_5848685
25,93
3F_4940898
25,94
PMS-30_L1
27,16
3F_5323005
27,65
3F_7210137
28,79
3A_6687690
29,14
3F_6780052
29,39
EPDCU3-083_L7
29,61
3F_7283430
29,73
EPDCU3-083_L6
30,98
3A_6566641
31,34
3F_7812427
31,99
3F_7626706
32,02
3A_8522819
32,30
4F_20442276
32,40
3A_9729116
33,72
3F_12188774
35,16
3A_16342197
36,38
3F_12375363
36,63
3A_12474678
36,83
3F_17994254
37,01
3F_12675693
37,04
3A_13001555
37,27
3F_18450567
37,51
3F_13369328
37,76
3A_10626205
39,79
3A_12500413
40,08
3F_12539794
41,54
PC2_3F
3A_1512481
0,00
3A_1307790
0,68
3F_1953269
1,36
3A_1720554
2,44
3A_511746
4,14
3A_1656893
4,67
3A_1810714
4,83
3F_1182045
3A_2008113
4,88
3F_1356250
5,15
3A_1414476
5,16
3A_2307153
5,31
3F_2203771
3F_3072067
5,32
3A_1663209
5,40
8F_1247883
5,72
PMS-30_L3
6,21
UCDP97-403_L1
6,56
3A_3409651
6,78
3A_3515662
3A_3196604
6,95
3A_2615230
6,96
3A_3509528
7,48
3F_4940898
10,06
3A_5848685
10,13
PMS-30_L1
11,03
3F_4633547
11,21
3F_5323005
11,81
BPPCT039_L1
12,33
EPDCU3-083_L4
12,89
3F_7210137
13,54
3A_6687690
13,75
3F_7283430
14,08
EPDCU3-083_L7
14,28
3F_6780052
14,61
3F_7626706
16,13
4F_20442276
16,49
3A_8522819
16,60
3F_7812427
16,83
3A_6566641
17,29
BPPCT009_L1
18,11
3F_12188774
19,46
3A_16342197
21,05
3F_12375363
21,31
3A_12474678
21,58
3F_12675693
21,77
3F_17994254
21,79
3A_13001555
22,01
3F_18450567
22,17
3F_13369328
22,45
3A_10626205
24,75
3A_12500413
25,10
3F_12539794
25,89
3F_18446535
27,32
3A_13715535
29,46
3A_19310781
30,02
3A_20255605
31,84
3F_15171728
32,50
3F_16122412
34,38
3A_21024386
36,08
3A_22297749
38,52
3F_17534527
40,04
8F_2411873
40,50
3F_17988706
40,54
3F_18467711
41,78
3A_17073831
43,49
1F_27534222
47,77
3F_20012004
49,98
3A_20357254
52,63
3A_20736561
53,22
3A_26713050
57,79
3F_20295529
62,66
3

## Slide 4
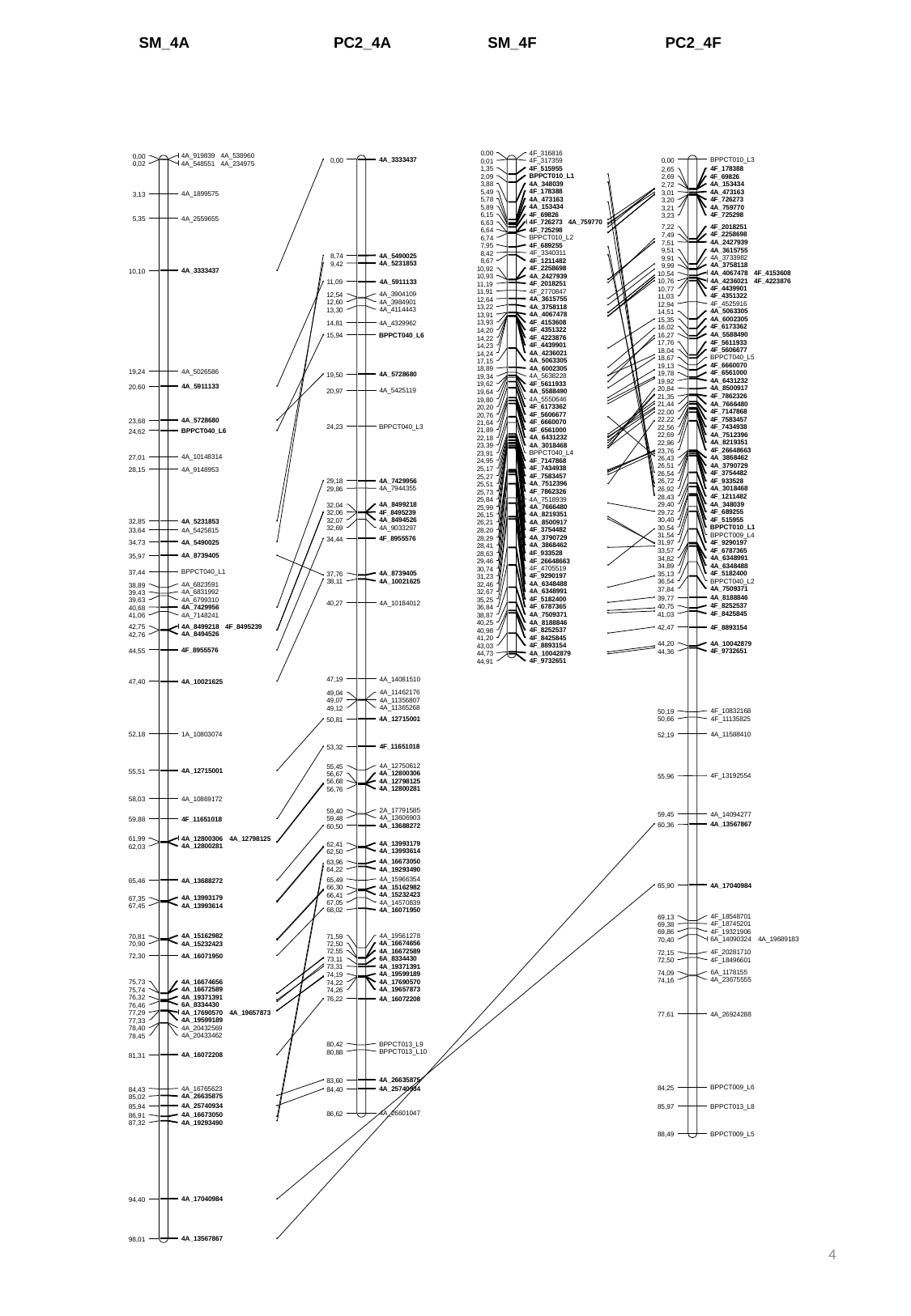

SM_4A
4A_919839
4A_538960
0,00
4A_548551
4A_234975
0,02
4A_1899575
3,13
4A_2559655
5,35
4A_3333437
10,10
4A_5026586
19,24
4A_5911133
20,60
4A_5728680
23,68
BPPCT040_L6
24,62
4A_10148314
27,01
4A_9148953
28,15
4A_5231853
32,85
4A_5425815
33,64
4A_5490025
34,73
4A_8739405
35,97
BPPCT040_L1
37,44
4A_6823591
38,89
4A_6831992
39,43
4A_6799310
39,63
4A_7429956
40,68
4A_7148241
41,06
4A_8499218
4F_8495239
42,75
4A_8494526
42,76
4F_8955576
44,55
4A_10021625
47,40
1A_10803074
52,18
4A_12715001
55,51
4A_10869172
58,03
4F_11651018
59,88
4A_12800306
4A_12798125
61,99
4A_12800281
62,03
4A_13688272
65,46
4A_13993179
67,35
4A_13993614
67,45
4A_15162982
70,81
4A_15232423
70,90
4A_16071950
72,30
4A_16674656
75,73
4A_16672589
75,74
4A_19371391
76,32
6A_8334430
76,46
4A_17690570
4A_19657873
77,29
4A_19599189
77,33
4A_20432569
78,40
4A_20433462
78,45
4A_16072208
81,31
4A_16765623
84,43
4A_26635875
85,02
4A_25740934
85,94
4A_16673050
86,91
4A_19293490
87,32
4A_17040984
94,40
4A_13567867
98,01
PC2_4A
4A_3333437
0,00
4A_5490025
8,74
4A_5231853
9,42
4A_5911133
11,09
4A_3904109
12,54
4A_3984901
12,60
4A_4114443
13,30
4A_4329962
14,81
BPPCT040_L6
15,94
4A_5728680
19,50
4A_5425119
20,97
BPPCT040_L3
24,23
4A_7429956
29,18
4A_7944355
29,86
4A_8499218
32,04
4F_8495239
32,06
4A_8494526
32,07
4A_9033297
32,69
4F_8955576
34,44
4A_8739405
37,76
4A_10021625
38,11
4A_10184012
40,27
4A_14081510
47,19
4A_11462176
49,04
4A_11356807
49,07
4A_11365268
49,12
4A_12715001
50,81
4F_11651018
53,32
4A_12750612
55,45
4A_12800306
56,67
4A_12798125
56,68
4A_12800281
56,76
2A_17791585
59,40
4A_13606903
59,48
4A_13688272
60,50
4A_13993179
62,41
4A_13993614
62,50
4A_16673050
63,96
4A_19293490
64,22
4A_15966354
65,49
4A_15162982
66,30
4A_15232423
66,41
4A_14570839
67,05
4A_16071950
68,02
4A_19561278
71,59
4A_16674656
72,50
4A_16672589
72,55
6A_8334430
73,11
4A_19371391
73,31
4A_19599189
74,19
4A_17690570
74,22
4A_19657873
74,26
4A_16072208
76,22
BPPCT013_L9
80,42
BPPCT013_L10
80,88
4A_26635875
83,60
4A_25740934
84,40
4A_26601047
86,62
SM_4F
4F_316816
0,00
4F_317359
0,01
4F_515955
1,35
BPPCT010_L1
2,09
4A_348039
3,88
4F_178388
5,49
4A_473163
5,78
4A_153434
5,89
4F_69826
6,15
4F_726273
4A_759770
6,63
4F_725298
6,64
BPPCT010_L2
6,74
4F_689255
7,95
4F_3340311
8,42
4F_1211482
8,67
4F_2258698
10,92
4A_2427939
10,93
4F_2018251
11,19
4F_2770847
11,91
4A_3615755
12,64
4A_3758118
13,22
4A_4067478
13,91
4F_4153608
13,93
4F_4351322
14,20
4F_4223876
14,22
4F_4439901
14,23
4A_4236021
14,24
4A_5063305
17,15
4A_6002305
18,89
4A_5638228
19,34
4F_5611933
19,62
4A_5588490
19,64
4A_5550646
19,80
4F_6173362
20,20
4F_5606677
20,76
4F_6660070
21,64
4F_6561000
21,89
4A_6431232
22,18
4A_3018468
23,39
BPPCT040_L4
23,91
4F_7147868
24,95
4F_7434938
25,17
4F_7583457
25,27
4A_7512396
25,51
4F_7862326
25,73
4A_7518939
25,84
4A_7666480
25,99
4A_8219351
26,15
4A_8500917
26,21
4F_3754482
28,20
4A_3790729
28,29
4A_3868462
28,41
4F_933528
28,63
4F_26648663
29,46
4F_4705519
30,74
4F_9290197
31,23
4A_6348488
32,46
4A_6348991
32,67
4F_5182400
35,25
4F_6787365
36,84
4A_7509371
38,87
4A_8188846
40,25
4F_8252537
40,98
4F_8425845
41,20
4F_8893154
43,03
4A_10042879
44,73
4F_9732651
44,91
PC2_4F
BPPCT010_L3
0,00
4F_178388
2,65
4F_69826
2,69
4A_153434
2,72
4A_473163
3,01
4F_726273
3,20
4A_759770
3,21
4F_725298
3,23
4F_2018251
7,22
4F_2258698
7,49
4A_2427939
7,51
4A_3615755
9,51
4A_3733982
9,91
4A_3758118
9,99
4A_4067478
4F_4153608
10,54
4A_4236021
4F_4223876
10,76
4F_4439901
10,77
4F_4351322
11,03
4F_4525916
12,94
4A_5063305
14,51
4A_6002305
15,35
4F_6173362
16,02
4A_5588490
16,27
4F_5611933
17,76
4F_5606677
18,04
BPPCT040_L5
18,67
4F_6660070
19,13
4F_6561000
19,78
4A_6431232
19,92
4A_8500917
20,84
4F_7862326
21,35
4A_7666480
21,44
4F_7147868
22,00
4F_7583457
22,22
4F_7434938
22,56
4A_7512396
22,69
4A_8219351
22,96
4F_26648663
23,76
4A_3868462
26,43
4A_3790729
26,51
4F_3754482
26,54
4F_933528
26,72
4A_3018468
26,92
4F_1211482
28,43
4A_348039
29,40
4F_689255
29,72
4F_515955
30,40
BPPCT010_L1
30,54
BPPCT009_L4
31,54
4F_9290197
31,97
4F_6787365
33,57
4A_6348991
34,82
4A_6348488
34,89
4F_5182400
35,13
BPPCT040_L2
36,54
4A_7509371
37,84
4A_8188846
39,77
4F_8252537
40,75
4F_8425845
41,03
4F_8893154
42,47
4A_10042879
44,20
4F_9732651
44,36
4F_10832168
50,19
4F_11135825
50,66
4A_11588410
52,19
4F_13192554
55,96
4A_14094277
59,45
4A_13567867
60,36
4A_17040984
65,90
4F_18548701
69,13
4F_18745201
69,38
4F_19321906
69,86
6A_14090324
4A_19689183
70,40
4F_20281710
72,15
4F_18496601
72,50
6A_1178155
74,09
4A_23675555
74,16
4A_26924288
77,61
BPPCT009_L6
84,25
BPPCT013_L8
85,97
BPPCT009_L5
88,49
4

## Slide 5
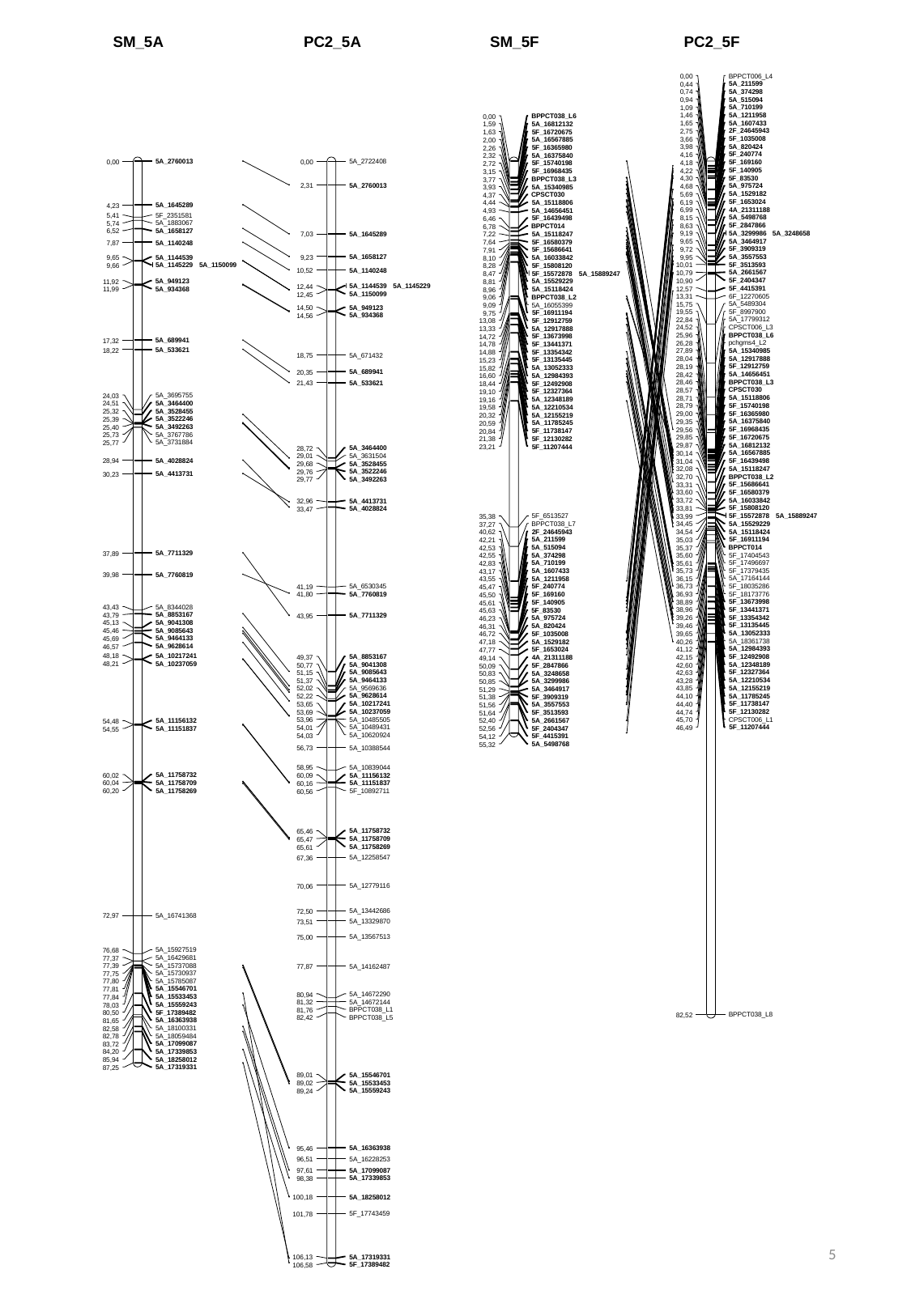

SM_5A
5A_2760013
0,00
5A_1645289
4,23
5F_2351581
5,41
5A_1883067
5,74
5A_1658127
6,52
5A_1140248
7,87
5A_1144539
9,65
5A_1145229
5A_1150099
9,66
5A_949123
11,92
5A_934368
11,99
5A_689941
17,32
5A_533621
18,22
5A_3695755
24,03
5A_3464400
24,51
5A_3528455
25,32
5A_3522246
25,39
5A_3492263
25,40
5A_3767786
25,73
5A_3731884
25,77
5A_4028824
28,94
5A_4413731
30,23
5A_7711329
37,89
5A_7760819
39,98
5A_8344028
43,43
5A_8853167
43,79
5A_9041308
45,13
5A_9085643
45,46
5A_9464133
45,69
5A_9628614
46,57
5A_10217241
48,18
5A_10237059
48,21
5A_11156132
54,48
5A_11151837
54,55
5A_11758732
60,02
5A_11758709
60,04
5A_11758269
60,20
5A_16741368
72,97
5A_15927519
76,68
5A_16429681
77,37
5A_15737088
77,39
5A_15730937
77,75
5A_15785087
77,80
5A_15546701
77,81
5A_15533453
77,84
5A_15559243
78,03
5F_17389482
80,50
5A_16363938
81,65
5A_18100331
82,58
5A_18059484
82,78
5A_17099087
83,72
5A_17339853
84,20
5A_18258012
85,94
5A_17319331
87,25
PC2_5A
5A_2722408
0,00
5A_2760013
2,31
5A_1645289
7,03
5A_1658127
9,23
5A_1140248
10,52
5A_1144539
5A_1145229
12,44
5A_1150099
12,45
5A_949123
14,50
5A_934368
14,56
5A_671432
18,75
5A_689941
20,35
5A_533621
21,43
5A_3464400
28,72
5A_3631504
29,01
5A_3528455
29,68
5A_3522246
29,76
5A_3492263
29,77
5A_4413731
32,96
5A_4028824
33,47
5A_6530345
41,19
5A_7760819
41,80
5A_7711329
43,95
5A_8853167
49,37
5A_9041308
50,77
5A_9085643
51,15
5A_9464133
51,37
5A_9569636
52,02
5A_9628614
52,22
5A_10217241
53,65
5A_10237059
53,69
5A_10485505
53,96
5A_10489431
54,01
5A_10620924
54,03
5A_10388544
56,73
5A_10839044
58,95
5A_11156132
60,09
5A_11151837
60,16
5F_10892711
60,56
5A_11758732
65,46
5A_11758709
65,47
5A_11758269
65,61
5A_12258547
67,36
5A_12779116
70,06
5A_13442686
72,50
5A_13329870
73,51
5A_13567513
75,00
5A_14162487
77,87
5A_14672290
80,94
5A_14672144
81,32
BPPCT038_L1
81,76
BPPCT038_L5
82,42
5A_15546701
89,01
5A_15533453
89,02
5A_15559243
89,24
5A_16363938
95,46
5A_16228253
96,51
5A_17099087
97,61
5A_17339853
98,38
5A_18258012
100,18
5F_17743459
101,78
5A_17319331
106,13
5F_17389482
106,58
SM_5F
BPPCT038_L6
0,00
5A_16812132
1,59
5F_16720675
1,63
5A_16567885
2,00
5F_16365980
2,26
5A_16375840
2,32
5F_15740198
2,72
5F_16968435
3,15
BPPCT038_L3
3,77
5A_15340985
3,93
CPSCT030
4,37
5A_15118806
4,44
5A_14656451
4,93
5F_16439498
6,46
BPPCT014
6,78
5A_15118247
7,22
5F_16580379
7,64
5F_15686641
7,91
5A_16033842
8,10
5F_15808120
8,28
5F_15572878
5A_15889247
8,47
5A_15529229
8,81
5A_15118424
8,96
BPPCT038_L2
9,06
5A_16055399
9,09
5F_16911194
9,75
5F_12912759
13,08
5A_12917888
13,33
5F_13673998
14,72
5F_13441371
14,78
5F_13354342
14,88
5F_13135445
15,23
5A_13052333
15,82
5A_12984393
16,60
5F_12492908
18,44
5F_12327364
19,10
5A_12348189
19,16
5A_12210534
19,58
5A_12155219
20,32
5A_11785245
20,59
5F_11738147
20,84
5F_12130282
21,38
5F_11207444
23,21
5F_6513527
35,38
BPPCT038_L7
37,27
2F_24645943
40,62
5A_211599
42,21
5A_515094
42,53
5A_374298
42,55
5A_710199
42,83
5A_1607433
43,17
5A_1211958
43,55
5F_240774
45,47
5F_169160
45,50
5F_140905
45,61
5F_83530
45,63
5A_975724
46,23
5A_820424
46,31
5F_1035008
46,72
5A_1529182
47,18
5F_1653024
47,77
4A_21311188
49,14
5F_2847866
50,09
5A_3248658
50,83
5A_3299986
50,85
5A_3464917
51,29
5F_3909319
51,38
5A_3557553
51,56
5F_3513593
51,64
5A_2661567
52,40
5F_2404347
52,56
5F_4415391
54,12
5A_5498768
55,32
PC2_5F
BPPCT006_L4
0,00
5A_211599
0,44
5A_374298
0,74
5A_515094
0,94
5A_710199
1,09
5A_1211958
1,46
5A_1607433
1,65
2F_24645943
2,75
5F_1035008
3,66
5A_820424
3,98
5F_240774
4,16
5F_169160
4,18
5F_140905
4,22
5F_83530
4,30
5A_975724
4,68
5A_1529182
5,69
5F_1653024
6,19
4A_21311188
6,99
5A_5498768
8,15
5F_2847866
8,63
5A_3299986
5A_3248658
9,19
5A_3464917
9,65
5F_3909319
9,72
5A_3557553
9,95
5F_3513593
10,01
5A_2661567
10,79
5F_2404347
10,90
5F_4415391
12,57
6F_12270605
13,31
5A_5489304
15,75
5F_8997900
19,55
5A_17799312
22,84
CPSCT006_L3
24,52
BPPCT038_L6
25,96
pchgms4_L2
26,28
5A_15340985
27,89
5A_12917888
28,04
5F_12912759
28,19
5A_14656451
28,42
BPPCT038_L3
28,46
CPSCT030
28,57
5A_15118806
28,71
5F_15740198
28,79
5F_16365980
29,00
5A_16375840
29,35
5F_16968435
29,56
5F_16720675
29,85
5A_16812132
29,87
5A_16567885
30,14
5F_16439498
31,04
5A_15118247
32,08
BPPCT038_L2
32,70
5F_15686641
33,31
5F_16580379
33,60
5A_16033842
33,72
5F_15808120
33,81
5F_15572878
5A_15889247
33,99
5A_15529229
34,45
5A_15118424
34,54
5F_16911194
35,03
BPPCT014
35,37
5F_17404543
35,60
5F_17496697
35,61
5F_17379435
35,73
5A_17164144
36,15
5F_18035286
36,73
5F_18173776
36,93
5F_13673998
38,89
5F_13441371
38,96
5F_13354342
39,26
5F_13135445
39,46
5A_13052333
39,65
5A_18361738
40,26
5A_12984393
41,12
5F_12492908
42,15
5A_12348189
42,60
5F_12327364
42,63
5A_12210534
43,28
5A_12155219
43,85
5A_11785245
44,10
5F_11738147
44,40
5F_12130282
44,74
CPSCT006_L1
45,70
5F_11207444
46,49
BPPCT038_L8
82,52
5

## Slide 6
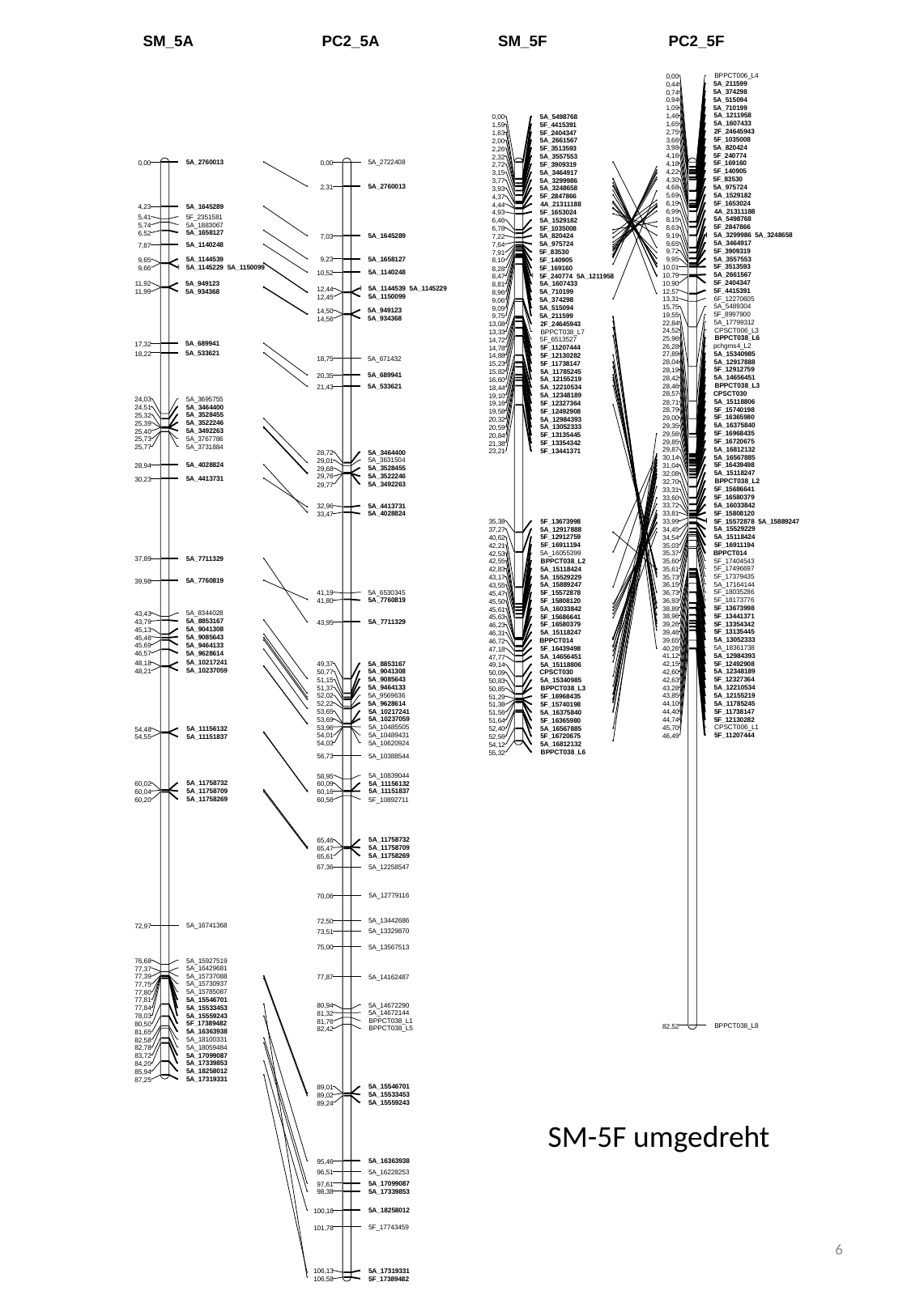

SM_5A
5A_2760013
0,00
5A_1645289
4,23
5F_2351581
5,41
5A_1883067
5,74
5A_1658127
6,52
5A_1140248
7,87
5A_1144539
9,65
5A_1145229
5A_1150099
9,66
5A_949123
11,92
5A_934368
11,99
5A_689941
17,32
5A_533621
18,22
5A_3695755
24,03
5A_3464400
24,51
5A_3528455
25,32
5A_3522246
25,39
5A_3492263
25,40
5A_3767786
25,73
5A_3731884
25,77
5A_4028824
28,94
5A_4413731
30,23
5A_7711329
37,89
5A_7760819
39,98
5A_8344028
43,43
5A_8853167
43,79
5A_9041308
45,13
5A_9085643
45,46
5A_9464133
45,69
5A_9628614
46,57
5A_10217241
48,18
5A_10237059
48,21
5A_11156132
54,48
5A_11151837
54,55
5A_11758732
60,02
5A_11758709
60,04
5A_11758269
60,20
5A_16741368
72,97
5A_15927519
76,68
5A_16429681
77,37
5A_15737088
77,39
5A_15730937
77,75
5A_15785087
77,80
5A_15546701
77,81
5A_15533453
77,84
5A_15559243
78,03
5F_17389482
80,50
5A_16363938
81,65
5A_18100331
82,58
5A_18059484
82,78
5A_17099087
83,72
5A_17339853
84,20
5A_18258012
85,94
5A_17319331
87,25
PC2_5A
5A_2722408
0,00
5A_2760013
2,31
5A_1645289
7,03
5A_1658127
9,23
5A_1140248
10,52
5A_1144539
5A_1145229
12,44
5A_1150099
12,45
5A_949123
14,50
5A_934368
14,56
5A_671432
18,75
5A_689941
20,35
5A_533621
21,43
5A_3464400
28,72
5A_3631504
29,01
5A_3528455
29,68
5A_3522246
29,76
5A_3492263
29,77
5A_4413731
32,96
5A_4028824
33,47
5A_6530345
41,19
5A_7760819
41,80
5A_7711329
43,95
5A_8853167
49,37
5A_9041308
50,77
5A_9085643
51,15
5A_9464133
51,37
5A_9569636
52,02
5A_9628614
52,22
5A_10217241
53,65
5A_10237059
53,69
5A_10485505
53,96
5A_10489431
54,01
5A_10620924
54,03
5A_10388544
56,73
5A_10839044
58,95
5A_11156132
60,09
5A_11151837
60,16
5F_10892711
60,56
5A_11758732
65,46
5A_11758709
65,47
5A_11758269
65,61
5A_12258547
67,36
5A_12779116
70,06
5A_13442686
72,50
5A_13329870
73,51
5A_13567513
75,00
5A_14162487
77,87
5A_14672290
80,94
5A_14672144
81,32
BPPCT038_L1
81,76
BPPCT038_L5
82,42
5A_15546701
89,01
5A_15533453
89,02
5A_15559243
89,24
5A_16363938
95,46
5A_16228253
96,51
5A_17099087
97,61
5A_17339853
98,38
5A_18258012
100,18
5F_17743459
101,78
5A_17319331
106,13
5F_17389482
106,58
SM_5F
5A_5498768
0,00
5F_4415391
1,59
5F_2404347
1,63
5A_2661567
2,00
5F_3513593
2,26
5A_3557553
2,32
5F_3909319
2,72
5A_3464917
3,15
5A_3299986
3,77
5A_3248658
3,93
5F_2847866
4,37
4A_21311188
4,44
5F_1653024
4,93
5A_1529182
6,46
5F_1035008
6,78
5A_820424
7,22
5A_975724
7,64
5F_83530
7,91
5F_140905
8,10
5F_169160
8,28
5F_240774
5A_1211958
8,47
5A_1607433
8,81
5A_710199
8,96
5A_374298
9,06
5A_515094
9,09
5A_211599
9,75
2F_24645943
13,08
BPPCT038_L7
13,33
5F_6513527
14,72
5F_11207444
14,78
5F_12130282
14,88
5F_11738147
15,23
5A_11785245
15,82
5A_12155219
16,60
5A_12210534
18,44
5A_12348189
19,10
5F_12327364
19,16
5F_12492908
19,58
5A_12984393
20,32
5A_13052333
20,59
5F_13135445
20,84
5F_13354342
21,38
5F_13441371
23,21
5F_13673998
35,38
5A_12917888
37,27
5F_12912759
40,62
5F_16911194
42,21
5A_16055399
42,53
BPPCT038_L2
42,55
5A_15118424
42,83
5A_15529229
43,17
5A_15889247
43,55
5F_15572878
45,47
5F_15808120
45,50
5A_16033842
45,61
5F_15686641
45,63
5F_16580379
46,23
5A_15118247
46,31
BPPCT014
46,72
5F_16439498
47,18
5A_14656451
47,77
5A_15118806
49,14
CPSCT030
50,09
5A_15340985
50,83
BPPCT038_L3
50,85
5F_16968435
51,29
5F_15740198
51,38
5A_16375840
51,56
5F_16365980
51,64
5A_16567885
52,40
5F_16720675
52,56
5A_16812132
54,12
BPPCT038_L6
55,32
PC2_5F
BPPCT006_L4
0,00
5A_211599
0,44
5A_374298
0,74
5A_515094
0,94
5A_710199
1,09
5A_1211958
1,46
5A_1607433
1,65
2F_24645943
2,75
5F_1035008
3,66
5A_820424
3,98
5F_240774
4,16
5F_169160
4,18
5F_140905
4,22
5F_83530
4,30
5A_975724
4,68
5A_1529182
5,69
5F_1653024
6,19
4A_21311188
6,99
5A_5498768
8,15
5F_2847866
8,63
5A_3299986
5A_3248658
9,19
5A_3464917
9,65
5F_3909319
9,72
5A_3557553
9,95
5F_3513593
10,01
5A_2661567
10,79
5F_2404347
10,90
5F_4415391
12,57
6F_12270605
13,31
5A_5489304
15,75
5F_8997900
19,55
5A_17799312
22,84
CPSCT006_L3
24,52
BPPCT038_L6
25,96
pchgms4_L2
26,28
5A_15340985
27,89
5A_12917888
28,04
5F_12912759
28,19
5A_14656451
28,42
BPPCT038_L3
28,46
CPSCT030
28,57
5A_15118806
28,71
5F_15740198
28,79
5F_16365980
29,00
5A_16375840
29,35
5F_16968435
29,56
5F_16720675
29,85
5A_16812132
29,87
5A_16567885
30,14
5F_16439498
31,04
5A_15118247
32,08
BPPCT038_L2
32,70
5F_15686641
33,31
5F_16580379
33,60
5A_16033842
33,72
5F_15808120
33,81
5F_15572878
5A_15889247
33,99
5A_15529229
34,45
5A_15118424
34,54
5F_16911194
35,03
BPPCT014
35,37
5F_17404543
35,60
5F_17496697
35,61
5F_17379435
35,73
5A_17164144
36,15
5F_18035286
36,73
5F_18173776
36,93
5F_13673998
38,89
5F_13441371
38,96
5F_13354342
39,26
5F_13135445
39,46
5A_13052333
39,65
5A_18361738
40,26
5A_12984393
41,12
5F_12492908
42,15
5A_12348189
42,60
5F_12327364
42,63
5A_12210534
43,28
5A_12155219
43,85
5A_11785245
44,10
5F_11738147
44,40
5F_12130282
44,74
CPSCT006_L1
45,70
5F_11207444
46,49
BPPCT038_L8
82,52
SM-5F umgedreht
6

## Slide 7
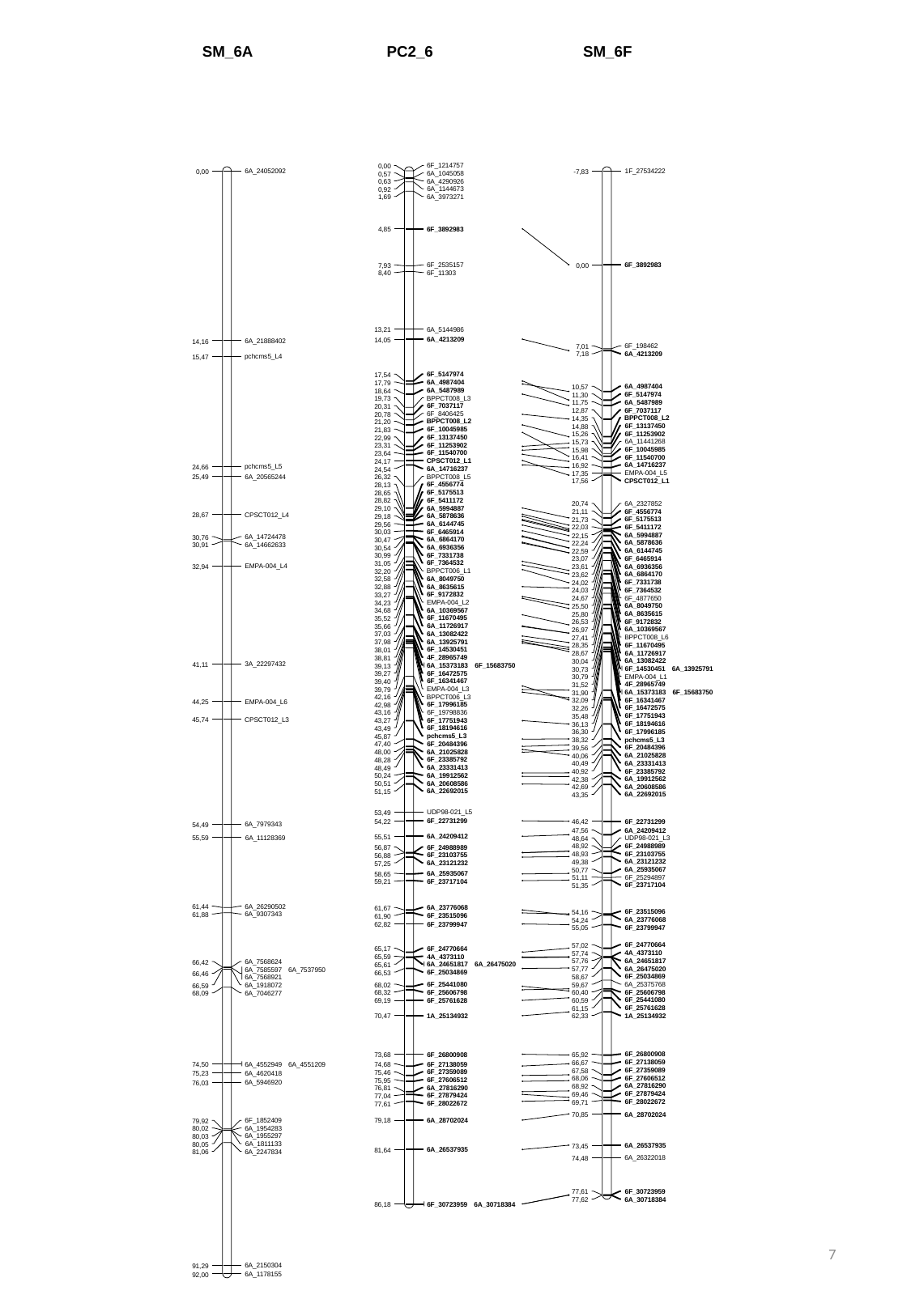

SM_6A
6A_24052092
0,00
6A_21888402
14,16
pchcms5_L4
15,47
pchcms5_L5
24,66
6A_20565244
25,49
CPSCT012_L4
28,67
6A_14724478
30,76
6A_14662633
30,91
EMPA-004_L4
32,94
3A_22297432
41,11
EMPA-004_L6
44,25
CPSCT012_L3
45,74
6A_7979343
54,49
6A_11128369
55,59
6A_26290502
61,44
6A_9307343
61,88
6A_7568624
66,42
6A_7585597
6A_7537950
66,46
6A_7568921
6A_1918072
66,59
6A_7046277
68,09
6A_4552949
6A_4551209
74,50
6A_4620418
75,23
6A_5946920
76,03
6F_1852409
79,92
6A_1954283
80,02
6A_1955297
80,03
6A_1811133
80,05
6A_2247834
81,06
6A_2150304
91,29
6A_1178155
92,00
PC2_6
6F_1214757
0,00
6A_1045058
0,57
6A_4290926
0,63
6A_1144673
0,92
6A_3973271
1,69
6F_3892983
4,85
6F_2535157
7,93
6F_11303
8,40
6A_5144986
13,21
6A_4213209
14,05
6F_5147974
17,54
6A_4987404
17,79
6A_5487989
18,64
BPPCT008_L3
19,73
6F_7037117
20,31
6F_8406425
20,78
BPPCT008_L2
21,20
6F_10045985
21,83
6F_13137450
22,99
6F_11253902
23,31
6F_11540700
23,64
CPSCT012_L1
24,17
6A_14716237
24,54
BPPCT008_L5
26,32
6F_4556774
28,13
6F_5175513
28,65
6F_5411172
28,82
6A_5994887
29,10
6A_5878636
29,18
6A_6144745
29,56
6F_6465914
30,03
6A_6864170
30,47
6A_6936356
30,54
6F_7331738
30,99
6F_7364532
31,05
BPPCT006_L1
32,20
6A_8049750
32,58
6A_8635615
32,88
6F_9172832
33,27
EMPA-004_L2
34,23
6A_10369567
34,68
6F_11670495
35,52
6A_11726917
35,66
6A_13082422
37,03
6A_13925791
37,98
6F_14530451
38,01
4F_28965749
38,81
6A_15373183
6F_15683750
39,13
6F_16472575
39,27
6F_16341467
39,40
EMPA-004_L3
39,79
BPPCT006_L3
42,16
6F_17996185
42,98
6F_19798836
43,16
6F_17751943
43,27
6F_18194616
43,49
pchcms5_L3
45,87
6F_20484396
47,40
6A_21025828
48,00
6F_23385792
48,28
6A_23331413
48,49
6A_19912562
50,24
6A_20608586
50,51
6A_22692015
51,15
UDP98-021_L5
53,49
6F_22731299
54,22
6A_24209412
55,51
6F_24988989
56,87
6F_23103755
56,88
6A_23121232
57,25
6A_25935067
58,65
6F_23717104
59,21
6A_23776068
61,67
6F_23515096
61,90
6F_23799947
62,82
6F_24770664
65,17
4A_4373110
65,59
6A_24651817
6A_26475020
65,61
6F_25034869
66,53
6F_25441080
68,02
6F_25606798
68,32
6F_25761628
69,19
1A_25134932
70,47
6F_26800908
73,68
6F_27138059
74,68
6F_27359089
75,46
6F_27606512
75,95
6A_27816290
76,81
6F_27879424
77,04
6F_28022672
77,61
6A_28702024
79,18
6A_26537935
81,64
6F_30723959
6A_30718384
86,18
SM_6F
1F_27534222
-7,83
6F_3892983
0,00
6F_198462
7,01
6A_4213209
7,18
6A_4987404
10,57
6F_5147974
11,30
6A_5487989
11,75
6F_7037117
12,87
BPPCT008_L2
14,35
6F_13137450
14,88
6F_11253902
15,26
6A_11441268
15,73
6F_10045985
15,98
6F_11540700
16,41
6A_14716237
16,92
EMPA-004_L5
17,35
CPSCT012_L1
17,56
6A_2327852
20,74
6F_4556774
21,11
6F_5175513
21,73
6F_5411172
22,03
6A_5994887
22,15
6A_5878636
22,24
6A_6144745
22,59
6F_6465914
23,07
6A_6936356
23,61
6A_6864170
23,62
6F_7331738
24,02
6F_7364532
24,03
6F_4877650
24,67
6A_8049750
25,50
6A_8635615
25,80
6F_9172832
26,53
6A_10369567
26,97
BPPCT008_L6
27,41
6F_11670495
28,35
6A_11726917
28,67
6A_13082422
30,04
6F_14530451
6A_13925791
30,73
EMPA-004_L1
30,79
4F_28965749
31,52
6A_15373183
6F_15683750
31,90
6F_16341467
32,09
6F_16472575
32,26
6F_17751943
35,48
6F_18194616
36,13
6F_17996185
36,30
pchcms5_L3
38,32
6F_20484396
39,56
6A_21025828
40,06
6A_23331413
40,49
6F_23385792
40,92
6A_19912562
42,38
6A_20608586
42,69
6A_22692015
43,35
6F_22731299
46,42
6A_24209412
47,56
UDP98-021_L3
48,64
6F_24988989
48,92
6F_23103755
48,93
6A_23121232
49,38
6A_25935067
50,77
6F_25294897
51,11
6F_23717104
51,35
6F_23515096
54,16
6A_23776068
54,24
6F_23799947
55,05
6F_24770664
57,02
4A_4373110
57,74
6A_24651817
57,76
6A_26475020
57,77
6F_25034869
58,67
6A_25375768
59,67
6F_25606798
60,40
6F_25441080
60,59
6F_25761628
61,15
1A_25134932
62,33
6F_26800908
65,92
6F_27138059
66,67
6F_27359089
67,58
6F_27606512
68,06
6A_27816290
68,92
6F_27879424
69,46
6F_28022672
69,71
6A_28702024
70,85
6A_26537935
73,45
6A_26322018
74,48
6F_30723959
77,61
6A_30718384
77,62
7

## Slide 8
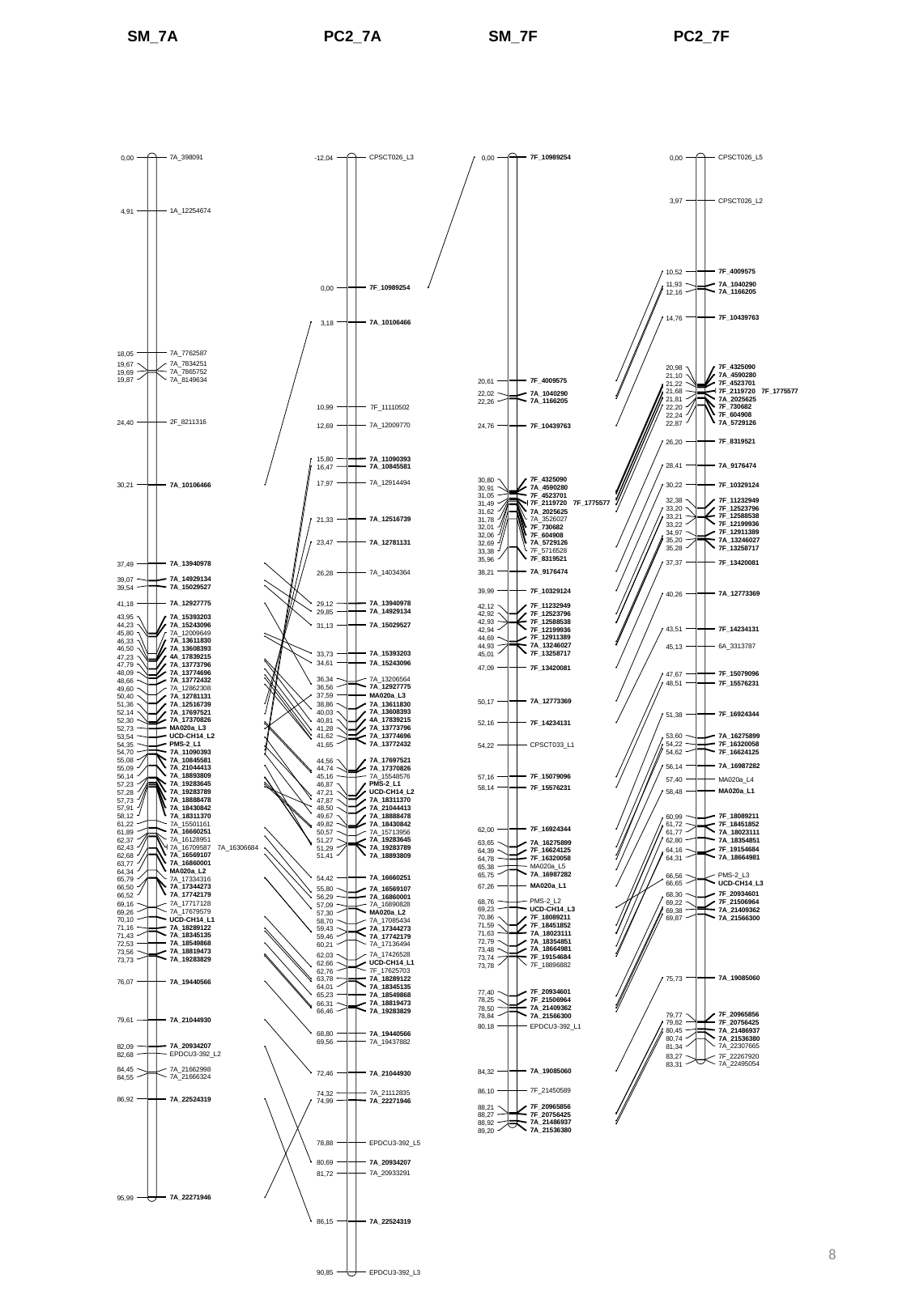

SM_7A
7A_398091
0,00
1A_12254674
4,91
7A_7762587
18,05
7A_7834251
19,67
7A_7865752
19,69
7A_8149634
19,87
2F_8211316
24,40
7A_10106466
30,21
7A_13940978
37,49
7A_14929134
39,07
7A_15029527
39,54
7A_12927775
41,18
7A_15393203
43,95
7A_15243096
44,23
7A_12009649
45,80
7A_13611830
46,33
7A_13608393
46,50
4A_17839215
47,23
7A_13773796
47,79
7A_13774696
48,09
7A_13772432
48,66
7A_12862308
49,60
7A_12781131
50,40
7A_12516739
51,36
7A_17697521
52,14
7A_17370826
52,30
MA020a_L3
52,73
UCD-CH14_L2
53,54
PMS-2_L1
54,35
7A_11090393
54,70
7A_10845581
55,08
7A_21044413
55,09
7A_18893809
56,14
7A_19283645
57,23
7A_19283789
57,28
7A_18888478
57,73
7A_18430842
57,91
7A_18311370
58,12
7A_15501161
61,22
7A_16660251
61,89
7A_16128951
62,37
7A_16709587
7A_16306684
62,43
7A_16569107
62,68
7A_16860001
63,77
MA020a_L2
64,34
7A_17334316
65,79
7A_17344273
66,50
7A_17742179
66,52
7A_17717128
69,16
7A_17679579
69,26
UCD-CH14_L1
70,10
7A_18289122
71,16
7A_18345135
71,43
7A_18549868
72,53
7A_18819473
73,56
7A_19283829
73,73
7A_19440566
76,07
7A_21044930
79,61
7A_20934207
82,09
EPDCU3-392_L2
82,68
7A_21662998
84,45
7A_21666324
84,55
7A_22524319
86,92
7A_22271946
95,99
PC2_7A
CPSCT026_L3
-12,04
7F_10989254
0,00
7A_10106466
3,18
7F_11110502
10,99
7A_12009770
12,69
7A_11090393
15,80
7A_10845581
16,47
7A_12914494
17,97
7A_12516739
21,33
7A_12781131
23,47
7A_14034364
26,28
7A_13940978
29,12
7A_14929134
29,85
7A_15029527
31,13
7A_15393203
33,73
7A_15243096
34,61
7A_13206564
36,34
7A_12927775
36,56
MA020a_L3
37,59
7A_13611830
38,86
7A_13608393
40,03
4A_17839215
40,81
7A_13773796
41,28
7A_13774696
41,62
7A_13772432
41,65
7A_17697521
44,56
7A_17370826
44,74
7A_15548576
45,16
PMS-2_L1
46,87
UCD-CH14_L2
47,21
7A_18311370
47,87
7A_21044413
48,50
7A_18888478
49,67
7A_18430842
49,82
7A_15713956
50,57
7A_19283645
51,27
7A_19283789
51,29
7A_18893809
51,41
7A_16660251
54,42
7A_16569107
55,80
7A_16860001
56,29
7A_16890828
57,09
MA020a_L2
57,30
7A_17085434
58,70
7A_17344273
59,43
7A_17742179
59,46
7A_17136494
60,21
7A_17426528
62,03
UCD-CH14_L1
62,66
7F_17625703
62,76
7A_18289122
63,78
7A_18345135
64,01
7A_18549868
65,23
7A_18819473
66,31
7A_19283829
66,46
7A_19440566
68,80
7A_19437882
69,56
7A_21044930
72,46
7A_21112835
74,32
7A_22271946
74,99
EPDCU3-392_L5
78,88
7A_20934207
80,69
7A_20933291
81,72
7A_22524319
86,15
EPDCU3-392_L3
90,85
SM_7F
7F_10989254
0,00
7F_4009575
20,61
7A_1040290
22,02
7A_1166205
22,26
7F_10439763
24,76
7F_4325090
30,80
7A_4590280
30,91
7F_4523701
31,05
7F_2119720
7F_1775577
31,49
7A_2025625
31,62
7A_3526027
31,78
7F_730682
32,01
7F_604908
32,06
7A_5729126
32,69
7F_5716528
33,38
7F_8319521
35,96
7A_9176474
38,21
7F_10329124
39,99
7F_11232949
42,12
7F_12523796
42,92
7F_12588538
42,93
7F_12199936
42,94
7F_12911389
44,69
7A_13246027
44,93
7F_13258717
45,01
7F_13420081
47,09
7A_12773369
50,17
7F_14234131
52,16
CPSCT033_L1
54,22
7F_15079096
57,16
7F_15576231
58,14
7F_16924344
62,00
7A_16275899
63,65
7F_16624125
64,39
7F_16320058
64,78
MA020a_L5
65,38
7A_16987282
65,75
MA020a_L1
67,26
PMS-2_L2
68,76
UCD-CH14_L3
69,23
7F_18089211
70,86
7F_18451852
71,59
7A_18023111
71,63
7A_18354851
72,79
7A_18664981
73,48
7F_19154684
73,74
7F_18896882
73,78
7F_20934601
77,40
7F_21506964
78,25
7A_21409362
78,50
7A_21566300
78,84
EPDCU3-392_L1
80,18
7A_19085060
84,32
7F_21450589
86,10
7F_20965856
88,21
7F_20756425
88,27
7A_21486937
88,92
7A_21536380
89,20
PC2_7F
CPSCT026_L5
0,00
CPSCT026_L2
3,97
7F_4009575
10,52
7A_1040290
11,93
7A_1166205
12,16
7F_10439763
14,76
7F_4325090
20,98
7A_4590280
21,10
7F_4523701
21,22
7F_2119720
7F_1775577
21,68
7A_2025625
21,81
7F_730682
22,20
7F_604908
22,24
7A_5729126
22,87
7F_8319521
26,20
7A_9176474
28,41
7F_10329124
30,22
7F_11232949
32,38
7F_12523796
33,20
7F_12588538
33,21
7F_12199936
33,22
7F_12911389
34,97
7A_13246027
35,20
7F_13258717
35,28
7F_13420081
37,37
7A_12773369
40,26
7F_14234131
43,51
6A_3313787
45,13
7F_15079096
47,67
7F_15576231
48,51
7F_16924344
51,38
7A_16275899
53,60
7F_16320058
54,22
7F_16624125
54,62
7A_16987282
56,14
MA020a_L4
57,40
MA020a_L1
58,48
7F_18089211
60,99
7F_18451852
61,72
7A_18023111
61,77
7A_18354851
62,80
7F_19154684
64,16
7A_18664981
64,31
PMS-2_L3
66,56
UCD-CH14_L3
66,65
7F_20934601
68,30
7F_21506964
69,22
7A_21409362
69,38
7A_21566300
69,87
7A_19085060
75,73
7F_20965856
79,77
7F_20756425
79,82
7A_21486937
80,45
7A_21536380
80,74
7A_22307665
81,34
7F_22267920
83,27
7A_22495054
83,31
8

## Slide 9
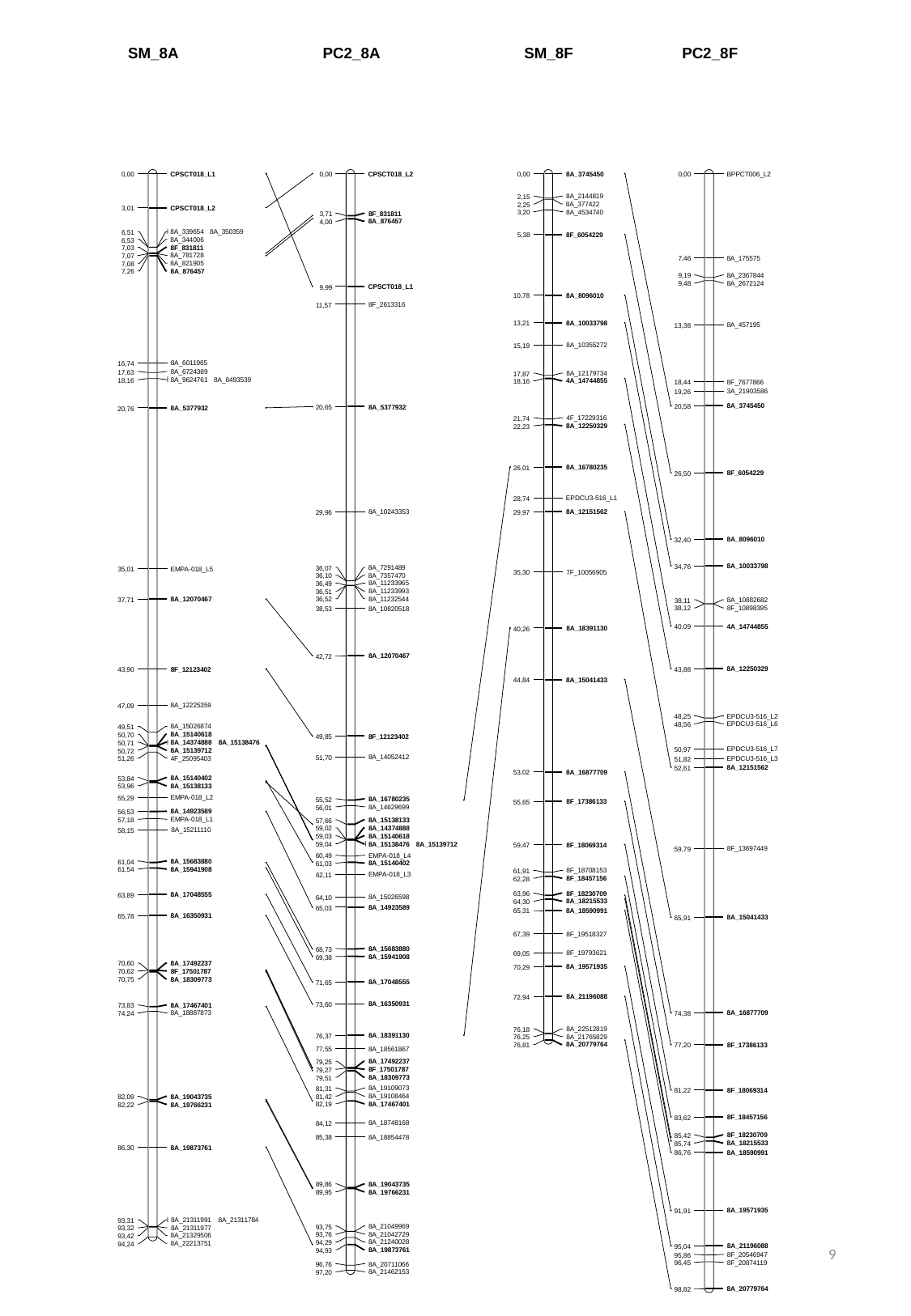

SM_8A
CPSCT018_L1
0,00
CPSCT018_L2
3,01
8A_339654
8A_350359
6,51
8A_344006
6,53
8F_831811
7,03
8A_781728
7,07
8A_821905
7,08
8A_876457
7,26
8A_6011965
16,74
8A_6724389
17,63
8A_9624761
8A_8493539
18,16
8A_5377932
20,76
EMPA-018_L5
35,01
8A_12070467
37,71
8F_12123402
43,90
8A_12225359
47,09
8A_15026874
49,51
8A_15140618
50,70
8A_14374888
8A_15138476
50,71
8A_15139712
50,72
4F_25095403
51,26
8A_15140402
53,84
8A_15138133
53,96
EMPA-018_L2
55,29
8A_14923589
56,53
EMPA-018_L1
57,18
8A_15211110
58,15
8A_15683880
61,04
8A_15941908
61,54
8A_17048555
63,89
8A_16350931
65,78
8A_17492237
70,60
8F_17501787
70,62
8A_18309773
70,75
8A_17467401
73,83
8A_18887873
74,24
8A_19043735
82,09
8A_19766231
82,22
8A_19873761
86,30
8A_21311991
8A_21311784
93,31
8A_21311977
93,32
8A_21329506
93,42
8A_22213751
94,24
PC2_8A
CPSCT018_L2
0,00
8F_831811
3,71
8A_876457
4,00
CPSCT018_L1
9,99
8F_2613316
11,57
8A_5377932
20,65
8A_10243353
29,96
8A_7291489
36,07
8A_7357470
36,10
8A_11233965
36,49
8A_11233993
36,51
8A_11232544
36,52
8A_10820518
38,53
8A_12070467
42,72
8F_12123402
49,85
8A_14052412
51,70
8A_16780235
55,52
8A_14629699
56,01
8A_15138133
57,66
8A_14374888
59,02
8A_15140618
59,03
8A_15138476
8A_15139712
59,04
EMPA-018_L4
60,49
8A_15140402
61,03
EMPA-018_L3
62,11
8A_15026598
64,10
8A_14923589
65,03
8A_15683880
68,73
8A_15941908
69,38
8A_17048555
71,65
8A_16350931
73,60
8A_18391130
76,37
8A_18561867
77,55
8A_17492237
79,25
8F_17501787
79,27
8A_18309773
79,51
8A_19109073
81,31
8A_19108464
81,42
8A_17467401
82,19
8A_18748168
84,12
8A_18854478
85,38
8A_19043735
89,86
8A_19766231
89,95
8A_21049969
93,75
8A_21042729
93,76
8A_21240028
94,29
8A_19873761
94,93
8A_20711066
96,76
8A_21462153
97,20
SM_8F
8A_3745450
0,00
8A_2144819
2,15
8A_377422
2,25
8A_4534740
3,20
8F_6054229
5,38
8A_8096010
10,78
8A_10033798
13,21
8A_10355272
15,19
8A_12179734
17,87
4A_14744855
18,16
4F_17229316
21,74
8A_12250329
22,23
8A_16780235
26,01
EPDCU3-516_L1
28,74
8A_12151562
29,97
7F_10056905
35,30
8A_18391130
40,26
8A_15041433
44,84
8A_16877709
53,02
8F_17386133
55,65
8F_18069314
59,47
8F_18708153
61,91
8F_18457156
62,28
8F_18230709
63,96
8A_18215533
64,30
8A_18590991
65,31
8F_19518327
67,39
8F_19793621
69,05
8A_19571935
70,29
8A_21196088
72,94
8A_22512819
76,18
8A_21765829
76,25
8A_20779764
76,81
PC2_8F
BPPCT006_L2
0,00
8A_175575
7,46
8A_2367844
9,19
8A_2672124
9,48
8A_457195
13,38
8F_7677866
18,44
3A_21903586
19,26
8A_3745450
20,58
8F_6054229
26,50
8A_8096010
32,40
8A_10033798
34,76
8A_10882682
38,11
8F_10898395
38,12
4A_14744855
40,09
8A_12250329
43,88
EPDCU3-516_L2
48,25
EPDCU3-516_L6
48,56
EPDCU3-516_L7
50,97
EPDCU3-516_L3
51,82
8A_12151562
52,61
8F_13697449
59,79
8A_15041433
65,91
8A_16877709
74,38
8F_17386133
77,20
8F_18069314
81,22
8F_18457156
83,62
8F_18230709
85,42
8A_18215533
85,74
8A_18590991
86,76
8A_19571935
91,91
8A_21196088
95,04
8F_20546947
95,86
8F_20874119
96,45
8A_20779764
98,82
9
