# Supplementary material for: Genetic architecture of cherry leaf spot (Blumeriella jaapii) resistance in sour cherry (Prunus cerasus L.) uncovered by QTL analyses in a biparental population genotyped with the 6 + 9 K SNP array
Source: Hortic Res. 2025 Feb 3;12(5):uhaf035. doi: 10.1093/hr/uhaf035 (PMC11992334; doi:10.1093/hr/uhaf035)
Supplement: Web_Material_uhaf035 [file web_material_uhaf035.zip › Table S1.docx]

| *P. cerasus* subgenome |  | 'Schattenmorelle' | | PC2 | |
| --- | --- | --- | --- | --- | --- |
|  | LG | no. markers assigned from | | | |
|  |  | *P. avium* | *P. fruticosa* | *P. avium* | *P. fruticosa* |
| *P.avium* | LG1 | 97 | 2 | 85 | 3 |
|  | LG2 | 53 | 2 | 51 | 4 |
|  | LG3 | 45 | 3 | 50 | 2 |
|  | LG4 | 53 | 3 | 51 | 3 |
|  | LG5 | 52 | 2 | 58 | 3 |
|  | LG6 | 26 | 1 | - | - |
|  | LG7 | 58 | 1 | 54 | 3 |
|  | LG8 | 38 | 4 | 42 | 4 |
| *P.fruticosa* | LG1 | 28 | 26 | 69 | 68 |
|  | LG2 | 32 | 39 | 32 | 38 |
|  | LG3 | 28 | 24 | 33 | 32 |
|  | LG4 | 28 | 37 | 35 | 41 |
|  | LG5 | 35 | 33 | 38 | 39 |
|  | LG6 | 37 | 47 | 38 | 48 |
|  | LG7 | 19 | 34 | 21 | 31 |
|  | LG8 | 22 | 10 | 19 | 10 |

**Table S1**. Number of markers originally obtained from species *P. avium* or *P. fruticosa* from the 6+9k SNP array.
