# Supplementary material for: Genetic architecture of cherry leaf spot (Blumeriella jaapii) resistance in sour cherry (Prunus cerasus L.) uncovered by QTL analyses in a biparental population genotyped with the 6 + 9 K SNP array
Source: Hortic Res. 2025 Feb 3;12(5):uhaf035. doi: 10.1093/hr/uhaf035 (PMC11992334; doi:10.1093/hr/uhaf035)
Supplement: Web_Material_uhaf035 [file web_material_uhaf035.zip › Table S2.docx]

**Table S2**. QTL mapping maximum LODs of crown defoliation scores

| Genotype | LG | 2021 | 2023 | avg. | CW thresholds |
| --- | --- | --- | --- | --- | --- |
| SM | 1a | **5.05** | 3.33 | ***3.99*** | 3.7 |
|  | 1f | **8.61** | **5.84** | **8.46** | 2.9 |
|  | 2a | 1.44 | 0.98 | 0.95 | 3.2 |
|  | *2f* | ***4.08*** | *1.95* | ***3.69*** | *3.2* |
|  | 3a | 1.64 | 1.79 | 1.59 | 3.3 |
|  | *3f* | *1.95* | ***5.57*** | ***4.2*** | *2.8* |
|  | 4a | 1.36 | 1.57 | 1.09 | 3.2 |
|  | 4f | 1.55 | 1.26 | 1.57 | 3.1 |
|  | 5a | 1.76 | 2.06 | 2.63 | 3 |
|  | 5f | 1.1 | 1.49 | 1.24 | 2.5 |
|  | 5f_2 | 1.59 | 2.28 | 2.09 | 2.6 |
|  | 6f | 1.25 | 4.18 | 2.15 | 3.3 |
|  | 7a | 2.12 | 1.51 | 1.56 | 3.4 |
|  | *7f* | 2.94 | ***3.91*** | ***3.93*** | 3 |
|  | 8a | 0.83 | 1.63 | 1.36 | 3 |
|  | 8f | 1.17 | 1.39 | 1.44 | 3.2 |
| GW thresholds |  | 4.9 | 4.5 | 4.8 |  |
| Pc2 | 1a | 1.97 | 2.05 | 1.74 | 3.5 |
|  | 1f | **10.04** | **6.6** | **9.2** | 4.2 |
|  | 2a | 2.48 | 1.67 | 1.89 | 3.2 |
|  | *2f* | ***4.07*** | *2.02* | ***3.68*** | *3.1* |
|  | 3a | 1.61 | 1.22 | 1.68 | 3.2 |
|  | *3f* | *2.84* | ***5.77*** | ***4.52*** | *3.2* |
|  | 4a | 1.14 | 1.26 | 0.92 | 3.3 |
|  | 4f | 3.2 | 1.25 | 2.08 | 3.3 |
|  | 5a | 1.75 | 2.19 | 2.51 | 3.1 |
|  | 5f | 1.67 | 1.82 | 1.64 | 4.1 |
|  | 6a | 1.2 | 0.81 | 4.27 | 8.6 |
|  | 6f | 1 | 1.54 | 1.04 | 3.2 |
|  | 7a | 2.28 | 2.82 | 1.42 | 3.1 |
|  | 7f | 1.81 | ***3.69*** | 2.69 | 3.2 |
|  | 8a | 0.6 | 1.69 | 1.26 | 3 |
|  | 8f | 1.03 | 1.4 | 1.45 | 3 |
| GW threshold |  | 9.8 | 6.6 | 8.7 |  |

LOD – logarithm of odd, LG – linkage group, CW - chromosome wide, GW – genome wide, SM – ‘Schattenmorelle’, Pc2 – sour cherry accession ‘Pc2’
